# Humanlikeness as design, anthropomorphism as inference: a conceptual framework for human–robot interaction

**DOI:** 10.3389/fcogn.2026.1786256

**Published:** 2026-06-03

**Authors:** Elizabeth K. Phillips, Ewart J. de Visser

**Affiliations:** 1 Applied Psychology and Autonomous Systems Laboratory, Department of Psychology, George Mason University, Fairfax, VA, United States; 2 United States Air Force Academy, Air Force Academy, CO, United States

**Keywords:** anthropomorphism, design forms, humanlike machines, humanlike robots, humanness, measurement

## Abstract

Humanlike robots are increasingly deployed in public and private settings, yet research and design efforts are hindered by persistent conceptual conflation between robots' humanlikeness and individuals' anthropomorphism, as well as by a hyperfocus on appearance-based definitions. This paper distinguishes humanlikeness as a multidimensional design property–the extent to which the design of an agent intentionally mimics human characteristics, traits, processes, or behaviors—from anthropomorphism as a psychological attribution process in which people ascribe human properties to non-human agents. We argue that treating these constructs as analytically distinct, while recognizing their reciprocal and dynamic interaction over time, enables more precise theorizing, clearer operationalization, and more actionable design guidance. To support cumulative interdisciplinary work, we situate both constructs within an integrated framework that organizes adjacent constructs (e.g., humanness, machinelikeness, animism, personification, and dehumanization) and provide curated measurement resources to facilitate principled study design and evaluation.

## 1 Introduction

The humanlike appearance of robots is a widely studied topic across multiple research communities. A substantial body of work demonstrates that robots' humanlike appearance systematically influences how they are perceived (Diel et al., 2021; Fink, 2012; Haring et al., 2018; Kätsyri et al., 2015; Kunold et al., 2023; Lohse et al., 2008; Rızvanoğlu et al., 2014). In general, the more humanlike a robot appears, the more humanlike qualities people attribute to it (Hegel et al., 2008; Sims et al., 2005). These attributions include perceptions of mind and cognitive capacities (Broadbent et al., 2013; Gray et al., 2023; Malle, 2019; Saltik et al., 2021; Zhao et al., 2019), as well as judgments of robots' intelligence (Sims et al., 2005), likeability (Castro-González et al., 2016), credibility (Siegel et al., 2009), trustworthiness (de Visser et al., 2016; Schaefer et al., 2012), and moral decision-making (Laakasuo et al., 2021; Malle et al., 2016; Momen et al., 2025; Tossell et al., 2025), among others.

As a result, the development of humanlike robots—robots with designed elements intended to mimic humans—has been accompanied by calls for caution. Concerns include the risk of fostering false expectations about robots' capabilities (Kwon et al., 2016; Phillips et al., 2017), facilitating unidirectional relationships or causing

emotional suffering (Scheutz, 2011, 2012), inherent deceptiveness (Danaher, 2020; Leong and Selinger, 2019; Rosero et al., 2024), and experiences of unease or disturbance (Kim et al., 2022; Mori, 1970; Mori et al., 2012). Others warn that people may project too many non-existent human qualities onto robots, potentially jeopardizing safe and appropriate use (Prather et al., 2020). In short, people will anthropomorphize them too much.

At the same time, meta-analytic evidence suggests that anthropomorphism can meaningfully benefit human–robot interaction, and that these effects vary substantially across task contexts, application domains, and design implementations, highlighting the importance of moderators in this process (Roesler et al., 2021). Importantly, anthropomorphism is widely understood as a default cognitive process through which humans naturally make sense of the world (Caporael and Heyes, 1997; Guthrie, 1997; Waytz et al., 2010a). From this perspective, it may be more productive to adopt approaches that embrace humanlike robot design as a known, and knowable, facilitator of anthropomorphism. Attempts to eliminate human anthropomorphism entirely in interactions with robots and other humanlike machines may be too difficult an endeavor, especially as the deployment of humanlike robots in public and private spaces is rapidly growing (Metz et al., 2025; Orrall, 2025).

**Figure 1:**
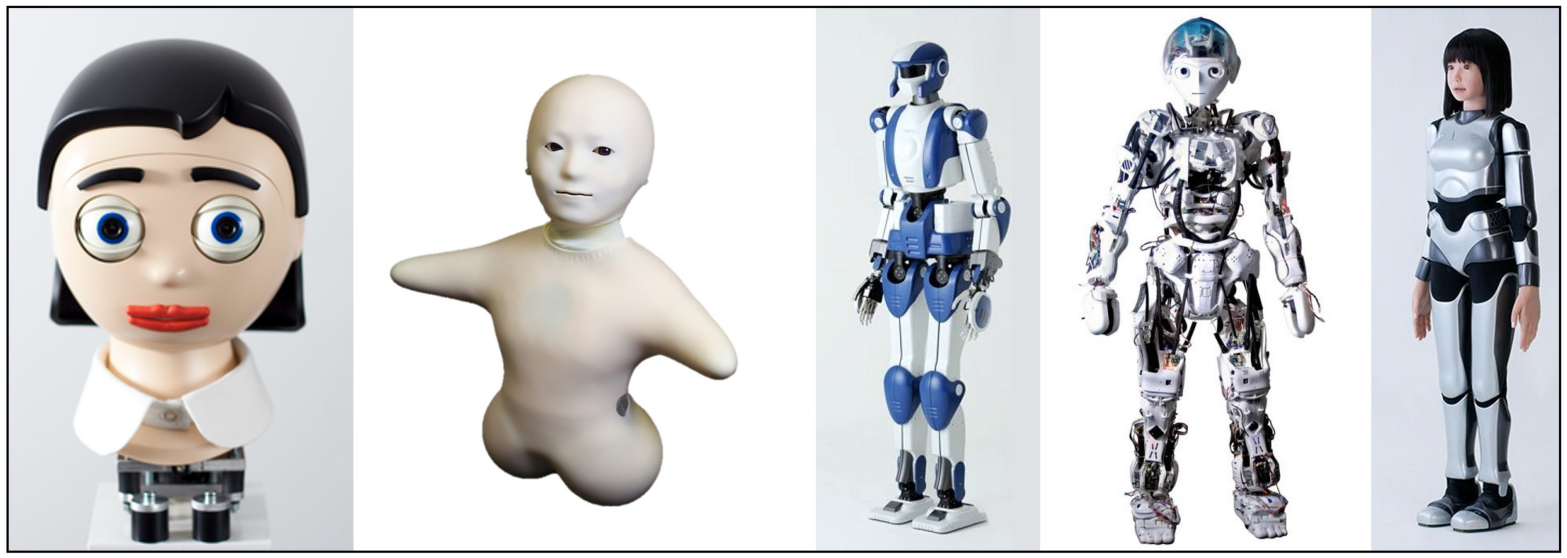
Examples of robots that have been labeled “humanlike,” “humanoid,” or “anthropomorphic” in the literature. The figure illustrates that robots often grouped under the same or similar design labels vary considerably in the number, type, and specificity of humanlike features present. In addition, these robots have been used in independent studies to examine the same or closely related attributional outcomes (Hegel et al., 2010; Ishiguro et al., 2013; Ferrari et al., 2016; Rosenthal-Von Der Pütten and Krämer, 2014), despite their substantial differences in humanlike design. Images obtained from the ABOT Database.

Despite this, several conceptual and methodological challenges to meeting this goal remain. For example, until recently, robot humanlikeness as a construct was theoretically underspecified. Often, robots described in the literature as “humanlike,” “humanoid,” or “anthropomorphic” varied considerably in their appearance, especially in the type, detail, and number of humanlike features present on the robot (see Figure 1). Further, the robot “humanlikeness” construct has also been treated as unidimensional, ranging on a spectrum from “non-humanlike,” or even “machinelike” (e.g., Bartneck et al., 2009), to “highly humanlike.” However, some robots can contain elements of both humanlikeness and machinelikeness simultaneously (Jayaraman et al., 2024; Kaplan et al., 2021), or may be very humanlike in some ways and not in others (e.g., possessing a very detailed face but lacking a body, Kim et al., 2022).

Because of the considerable variability in robots' physical appearance, researchers attempting to examine relationships between humanlike appearance and psycho, social, or behavioral outcomes of interest have had to rely upon two conventional experimental methodologies. The first is to designate relatively few robots with different appearances as examples of “mechanical,” “humanoid,” “humanlike,” or “android” and then compare human perceptions and anthropomorphism projections across them (Ferrari et al., 2016; Krach et al., 2008; Riek et al., 2009; Shimada et al., 2006). Figure 1, for example, illustrates robots that vary considerably in the number, type, and specificity of their humanlike design features, yet all have been labeled “humanlike,” “humanoid,” or “anthropomorphic” in their respective literatures. And these robots have been used in independent studies to examine the same or closely related attributional outcomes (Ferrari et al., 2016; Hegel et al., 2010; Ishiguro et al., 2013; Rosenthal-Von Der Pütten and Krämer, 2014).

The second has been to focus on a specific feature in robot models (e.g., the face; DiSalvo et al., 2002; Kalegina et al., 2018; Momen et al., 2024) and observe subsequent reactions to variations of this feature. Both approaches have heavily relied on researcher intuition and availability of robot exemplars, which has made it difficult to draw systematic conclusions across the literature about how specific features of humanlike design lead to the attribution of specific human characteristics.

Compounding this issue, the term *anthropomorphism* is also frequently used in the literature not only to describe the psychological process by which people attribute human qualities to robots, but also interchangeably with how humanlike a robot appears to be (i.e., its humanlikeness, see Abdi et al., 2022; Chung et al., 2022; Lin et al., 2025; Phillips et al., 2011; Yogeeswaran et al., 2016). Careful distinctions between anthropomorphism and humanlikeness are uncommon in much of the literature examining the consequences of robot appearance, especially in the human-robot and human-computer interaction research communities (HRI, HCI). For reviews from the HRI and HCI communities see Axelsson and Shevlin (2026), Damholdt et al. (2023), and Frazer (2022).

This conflation is problematic, as these constructs originate from distinct bodies of theory and inquiry across research, design, and engineering domains, each with unique conceptual and practical implications. Given the interdisciplinary nature of the research communities that study humanlike robots, failing to acknowledge these distinctions risks obscuring the richness of these constructs and undermines precision in communicating study designs and their findings. This in turn makes it difficult to place empirical results in appropriate context, extrapolate findings in practically meaningful ways (to design teams for instance), or integrate findings across studies and bodies of literature.

As such, it's difficult to know whether the humanlike design of robots–including which specific features of humanlike design–or people's attribution of human qualities to robots are the drivers of the beneficial effects uncovered by meta-analytic studies. The former would be relevant for providing prescriptive guidance for robot design, the latter relevant for meaningful interpretations of the consequences of that design. Thus, despite the considerable number of papers reporting on robot humanlikeness and anthropomorphism, there is a lack of theoretical coherence for how to treat humanlikeness, constituted by properties of a robot, to influence anthropomorphism in systematic, reliable, and meaningful ways. To use humanlikeness as a facilitator of anthropomorphism, a first step is that the constructs need to be well specified and treated as distinct.

### 1.1 Purpose

To address these challenges, the purpose of this paper is to:

- Clearly define the constructs of robot humanlikeness as a design property and anthropomorphism as a psychological attribution process, and articulate why these constructs should be treated as analytically distinct despite their frequent entanglement in research practice.
- Clarify how these constructs relate to one another across design, perception, and interaction, highlighting the implications of this relationship for robot design and evaluation.
- Situate humanlikeness and anthropomorphism within a broader conceptual landscape of related constructs that are commonly invoked, often implicitly or inconsistently, across human–robot interaction, psychology, philosophy, and design domains (e.g., humanness, dehumanization, animism, personification, and machinelikeness). By systematically defining and organizing these constructs, we present an integrated conceptual framework intended to serve as a shared theoretical and terminological resource for researchers working across disciplines (see Table 1).
- Support future empirical work by identifying and curating a set of existing measurement instruments and empirical approaches that have been used to capture humanlikeness, anthropomorphism, and closely related constructs in prior literature (see Table 2).

**Table 1:** Conceptual framework of humanlikeness, anthropomorphism, and related constructs organized by constructs related to conditions of being, properties of form, psychological processes, and broader representations.

Construct	Definition	Selected citations
Conditions		
Humanness (State of being)	The condition of being human, including characteristics and traits that define human nature–what is typical of being human–and human uniqueness–what distinguishes humans from animals.	Haslam, 2006; Haslam et al., 2013; Haslam and Loughnan, 2014; Leyens et al., 2007
Human uniqueness (Characteristics)	Human characteristics that distinguish humans from animals or automata and are thought to be uniquely human, including civility, refinement, moral sensibility, rationality, and maturity.	Haslam et al., 2013; Leyens et al., 2000
Human nature (Traits)	Human traits that are typical, fundamental, or common to being human but may also be shared with other species, including emotionality, warmth, openness, agency, and depth.	Haslam et al., 2013; Leyens et al., 2000
Humanity (Virtue)	Moral and normative standards that ground ethical obligations toward living beings by virtue of their status, independent of uniquely human traits.	Hill, 1980
Machineness (State of being)	The condition of being machine, including traits considered typical of machines but sometimes applied by analogy or contrast to biological entities, such as orderliness, efficiency, rigidity, conformity, passivity, fungibility, regularity, uniformity, and functional decomposition of parts.	Levy, 2014
Properties		
Humanlikeness (Design property) Anthropomorphic (Synonym)	The degree to which an object includes designed elements intentionally intended to mimic human characteristics, traits, features, processes, systems, and/or behaviors.	Phillips et al., 2018, 2017; DiSalvo et al., 2004, 2005; Rothstein et al., 2020
Animal-likeness (Design property) Zoomorphic (Synonym)	The degree to which an object includes designed elements intentionally intended to mimic animal characteristics, traits, features, processes, or behaviors.	Löffler et al., 2020
Machinelikeness (design property)	The degree to which an object includes designed characteristics that convey what is machinelike.	Levy, 2014
Robomorphic (design property)	Characteristics, features, or qualities found in robotic entities, including physical traits such as geometric form, action-related traits such as mechanical movement and replicable precision, and interaction-related traits such as goal-directedness.	Correia et al., 2024
Anthropomorphism (process)	The psychological process of attributing human properties, characteristics, or mental states to real or imagined non-human agents or objects.	Guthrie, 1997; Epley et al., 2007; Waytz et al., 2010a
Dehumanization (process)	The process of denying, rejecting, or replacing human characteristics by representing humans–often members of outgroups–as non-human agents or animals; the inverse of anthropomorphism.	Haslam, 2006; Haslam and Loughnan, 2014; Waytz and Epley, 2012
Mechanistic dehumanization (process)	A form of dehumanization in which humans are likened to objects or automata and denied traits of human nature such as warmth, emotion, or individuality.	Haslam, 2006; Waytz and Epley, 2012
Animalistic dehumanization (process)	A form of dehumanization in which humans are likened to animals and denied uniquely human traits such as civility, rationality, or moral sensibility.	Haslam, 2006; Waytz and Epley, 2012
Infrahumanization (process)	The process by which people attribute full humanness to their ingroup while denying human uniqueness or nature traits to outgroups.	Leyens et al., 2007
Zoomorphism (process)	The process of attributing animal characteristics to non-human agents or objects.	Nanay, 2021
Robomorphism (process)	The process of attributing robot traits to non-robotic entities, including humans, animals, or objects, based on perceived similarity to robots.	Correia et al., 2024
Technomorphism (process)	The process of attributing technological or machine characteristics to humans, often as an explanatory or interpretive tool.	Lum et al., 2011
Animism (process)	The attribution of life, agency, personality, or spirit to inanimate objects or natural phenomena.	Guthrie, 1997; Bird-David, 1999
Representations		
Personification (representation)	The representation of non-human entities or abstractions as persons, commonly used in rhetoric, art, literature, and metaphor.	Mark, 1987
Ethopoeia (representation)	A rhetorical practice involving the creation or impersonation of a character to evoke a human response to an entity.	Nass and Moon, 2000

**Table 2:** Summary of measurement tools for humanlikeness, anthropomorphism, and related constructs.

Measure	Description	Design, Trait, State	Citation
Humanlikeness			
Anthropomorphic roBOT database (ABOT)	Feature-presence scores for 16 humanlike appearance features, and 2 mechanical locomotion features organized into four appearance dimensions. Provides appearance profiles and an overall humanlikeness score for individual robots. Can also estimate humanlikeness for novel robot designs.	Design	Phillips et al., 2018
Single-item humanlikeness measure	Slider-based rating of overall perceived physical humanlikeness of a robot.	State	Phillips et al., 2018; Baird et al., 2018
Perceived Humanness Index	Six semantic differential scales capturing perceived humanlike appearance; developed as an alternative to the Godspeed anthropomorphism subscale.	State	Ho and MacDorman, 2010
Trait anthropomorphism			
Individual Differences in Anthropomorphism Questionnaire (IDAQ)	30-item measure of individual trait differences in the tendency to attribute human traits (emotions, consciousness, intentions, freewill) to technology, nature, and animals.	Trait	Waytz et al., 2010a
IDAQ—Child Form (IDAQ-CF)	12-item adaptation of the IDAQ for children, ages 5–9. Removes freewill items and uses age-appropriate response formats.	Trait	Severson and Lemm, 2016
Anthropomorphic tendencies scale (ATS)	78-item measure of dispositional tendencies to engage in anthropomorphism behaviors toward artifacts, animals, nature, and higher powers.	Trait	Chin et al., 2004, 2005
Anthropomorphism questionnaire	20-item measure assessing anthropomorphism tendencies, includes toy-directed and adult belief and behavior subscales.	Trait	Neave et al., 2015; Clutterbuck et al., 2022
State anthropomorphism			
Godspeed questionnaire series (GQS)	24-item semantic differential scale evaluating perceptions of a specific robot across five dimensions: anthropomorphism, animacy, likeability, perceived intelligence, and perceived safety. Note the anthropomorphism subscale includes both attribution and humanlike appearance items.	State	Bartneck et al., 2009
Human-robot interaction evaluation scale (HRIES)	16-item measure capturing trait attributions toward robots across four dimensions: sociability, animacy, agency, and disturbance. Authors report they adopted a multicomponent approach to anthropomorphism, explicitly integrating traits from social perception and dehumanization theory.	State	Spatola et al., 2021
Anthropomorphic response (AR) measure	10-item state measure organized into five sub-scales (Connection, Helpfulness, Trust, Empathy, and Satisfaction) capturing perceptions of and responses to AI agents during interaction. Note some sub-scales overlap conceptually with anthropomorphism outcomes.	State	Kim and Im, 2023
Machinelikeness			
Machine trait scale	105-item measure in which participants rate adjectives on their perceived machinelikeness. May be applicable to validating the constructs of machineness and machinelikeness, though further supporting empirical work is needed.	State	Kiesler and Goetz, 2002
Technomorphism			
Technomorphic Tendencies Scale (TTS)	30-item measure of individual differences in the tendency to apply technomorphism schemas when understanding human behavior and to perceive others as machinelike. May require additional theoretical and empirical work to distinguish between “technomorphic” and “technomorphism.”	Trait	Lum et al., 2011

The full list of items for each measure along with instructions for administration, response scaling, and scoring procedures (when available) are provided in the Supplementary materials accompanying this document.

## 2 Relationship between anthropomorphism and humanlikeness

First, we start by offering a comprehensive definition of the humanlikeness construct as applied to robots and autonomous machines as: *the degree to which an agent includes designed elements intended to mimic human characteristics, traits, features, processes, systems, and/or behaviors* (discussed in more detail below). By contrast, anthropomorphism is the psychological process of “attributing human properties, characteristics, or mental states to real or imagined non-human agents or objects (Epley et al., 2007 p. 865),” regardless of whether those properties, characteristics, or states were intended by the designer (see Section 3.1). Importantly, although humanlikeness reflects design intent and implementation, anthropomorphism reflects how humans interpret and make sense of what they perceive via attribution processes.

Although we argue that anthropomorphism and humanlikeness are distinct constructs, and should be treated as such in research, we also acknowledge that these constructs are not entirely orthogonal in practice. Their interrelationship emerges at the intersections between designs, human perceptions of those designs, attributions, and subsequent interactions over time. Human access to the world is mediated by constructed representations. People perceive and interpret the world through cognitive lenses shaped by prior experiences, expectations, and learned patterns. Anthropomorphism, particularly its cognitive determinants, influence these lenses, and in turn, how features of robot design are experienced and interpreted.

For example, as interactions with humanlike robots become more commonplace, both individual- and societal-level understandings of robots are likely to evolve (Kahn Jr et al., 2011), reshaping how humanlikeness cues are perceived and how attributions are applied. Early encounters with humanlike robots may prompt richer attributions due to novelty and limited category knowledge, whereas increased exposure and understanding of robotic mechanisms may constrain such attributions over time. Importantly, this shift will also not occur uniformly across people. Individuals differ in their tendency to anthropomorphize (Waytz et al., 2010a), meaning that some will continue to attribute human mental, emotional, or social qualities to robots more readily than others.

**Figure 2:**
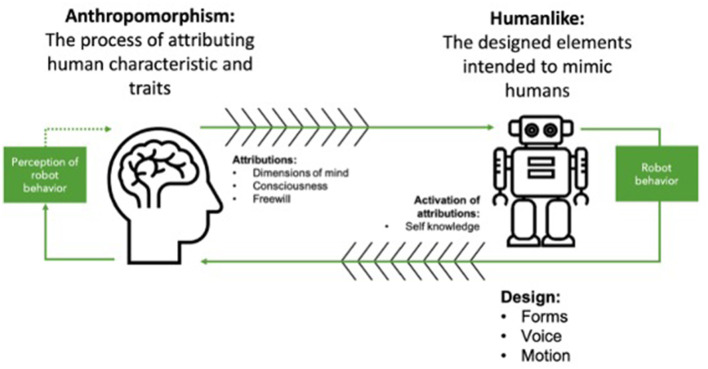
Depiction showing how humanlike robot design cues inform initial attributions, which are iteratively updated through interaction with the robot's behavior. Observers first encounter a robot's humanlike design features, which serve as perceptual cues that can prompt human attributions. These attributions then influence how users interpret the robot's behavior and evaluate interaction outcomes. Subsequent observation of and interaction with the robot's behavior provides additional information that can reinforce, revise, or constrain earlier interpretations, resulting in an ongoing iterative process linking design cues, attribution, and interaction experiences.

Figure 2 depicts the relationship between robot humanlikeness and anthropomorphism, illustrating how humanlike design cues shape initial perceptions and attributions, and how subsequent robot behavior feeds back into and updates those attributions over time. In this framework, observers first encounter a robot's humanlike design features, which guide perceptual interpretation and prompt the attribution of human characteristics. As the robot behaves and interacts with people, these behaviors provide new information that can reinforce, modify, or constrain earlier interpretations, resulting in an ongoing, iterative attribution process.

This relationship should not be interpreted as presenting robot design features and humanlike attribution as competing explanations of HRI outcomes. Rather, the two constructs occupy different positions within a coupled process. Humanlike design features function as design-level inputs that can cue humanlike attribution, while anthropomorphism functions as a proximal interpretive mechanism through which those cues often influence perceptions and interaction outcomes. In this sense, anthropomorphism likely operates as a mediating process linking humanlike design cues to human responses. At the same time, the strength and direction of these relationships are shaped by factors such as individual differences in tendency to anthropomorphize, prior experience with robots, task context, and the functional role of the system. Over repeated interaction, robot behavior may further reinforce, revise, or suppress earlier attributions, making the relationship between design features and human attributions dynamic.

Imagine a robot designed with “eyes” intended to mimic human eyes. They are located on a “head,” at the front of a “face,” with two (more or less) symmetrical orbits, which exhibit gaze patterns and saccadic movements akin to humans. Prior empirical studies suggest that humans will likely perceive these orbits as eyes (Phillips et al., 2018), its gaze as intentional (Admoni and Scassellati, 2017) and attribute social and cognitive dimensions of mind (Hietanen et al., 2026; Zhao et al., 2019).

Now imagine that when a person approaches this robot, the robot moves away. In one circumstance, the person may conclude that the robot is scared or frightened. An entirely different interpretation is that the robot is behaving politely by giving way to the person upon approach (Parasuraman and Miller, 2004). These two very different interpretations of the robot's behavior illustrate that two different social models can be applied to the robot's behavior, and each involve the attribution (i.e., anthropomorphism) of different human capacities (e.g., arousal, emotion, intention, or politeness, etiquette, norm competence).

Further, such attributions can vary as a function of prior knowledge and expertise. Observers familiar with robot design may infer that the “eyes” of this robot do not serve a functional purpose for robot-centric vision, but instead are designed specifically to engage the anthropomorphism process and elicit attributions that make humans feel comfortable, or to facilitate intuitive human interactions (e.g., socially evocative design; Breazeal, 2003). From this perspective, the designer may not attribute human qualities to the robot at all, even while anticipating that others will. But, which interpretation is “correct?” Fortunately, all can be simultaneously valid. The degree to which robots are perceived as humanlike is a function of robot design, how people experience that design, and what follows are attributions that come with it.

## 3 Why distinguishing humanlikeness and anthropomorphism matters for design and evaluation

The preceding discussion describes that robot humanlikeness and anthropomorphism are deeply entangled in lived interaction. Humanlike design cues shape perception, perception guides attribution, and those attributions evolve through experience with robots over time. However, it is precisely this entanglement that necessitates treating the two constructs as analytically distinct if we want to design for human-robot interaction. Treating humanlikeness as a multidimensional set of design inputs, and anthropomorphism as a non-monolithic set of attributional outcomes, transforms anthropomorphism from a potentially vague or problematic cognitive bias into a meaningful evaluation metric, and potentially a pathway for aspirational robot designs. This separation enables a design-oriented research stance in which specific humanlike features can be systematically linked to specific classes of attribution, supporting prediction, prescription, and principled evaluation in human–robot interaction (Kahn et al., 2007).

To illustrate, by intentionally manipulating humanlike features of robots, designers may be able to systematically amplify or attenuate the attribution of specific human qualities and characteristics, and align those attributions with the contexts in which robots are deployed. Prior work (Zhao et al., 2019) demonstrates that people attribute affective (e.g., positive and negative feelings), social (e.g., understanding others' minds), and moral (e.g., telling right from wrong) minded capacities to robots based solely on facial features, whereas attributions related to physical interaction with the world are more strongly tied to bodily features and the presence of manipulators such as arms, legs, and hands. These findings suggest that different dimensions of humanlikeness cue distinct classes of anthropomorphism inferences. A robot's humanlike appearance does not uniformly lead to global perceptions of a uniform “humanlike” mind. Viewed through this lens, humanlikeness becomes a tunable design parameter rather than a monolithic goal. In hospital contexts, for instance, designers may wish to suppress facial cues that invite attributions of moral cognition or moral agency (Kim et al., 2025), thereby reducing the risk that patients or staff interpret robots as autonomous moral decision-makers, while still leveraging other humanlike features to support effective, task-focused interactions.

If we treat humanlikeness as a design parameter, we can also treat anthropomorphism as an evaluation metric, for instance, by becoming a principled tool for evaluating the state-of-the-art in human-robot interaction design. For example, one core aim of HRI research is to create robots that function as effective interaction partners often achieved via human similarity. For example, (Breazeal, 2003) describes socially evocative robots as those designed to encourage anthropomorphism, and socially interfaced robots as those that employ humanlike social and communicative cues. Both classes of robots rely on humanlike design to facilitate interaction by leveraging social models of human-human interaction. When the constructs are distinguished, anthropomorphism can be treated not as a confound, but as an outcome measure. If people anthropomorphize a socially evocative robot in ways consistent with its design goals, then the design can be said to have achieved its intended interaction purpose.

Further, the intentional design of humanlikeness in robot features, behaviors, and other compositions and people's responses to them can be used to assess what we mean by “effective interactions.” For instance, we can ask, “Are our robots civil?,” “Which humanlike elements have been designed to create such civility in robots?”, “Have people subsequently attributed traits of civility?” “And ultimately, have people benefited from robot civility?” Robots designed to understand and exhibit politeness norms for behavior (i.e., a humanlike feature of their design), for instance, could be a means to facilitate the trait of “civility.” Civility is often characterized as a secondary trait of humanness, and its purposeful design reflects an aspirational move beyond surface-level humanlikeness toward more value-laden dimensions of humanlikeness (a burgeoning area of research in the HRI community; Williams, 2025; Winkle et al., 2023).

### 3.1 Inferential boundaries: a framework for interpreting human attribution responses

**Figure 3:**
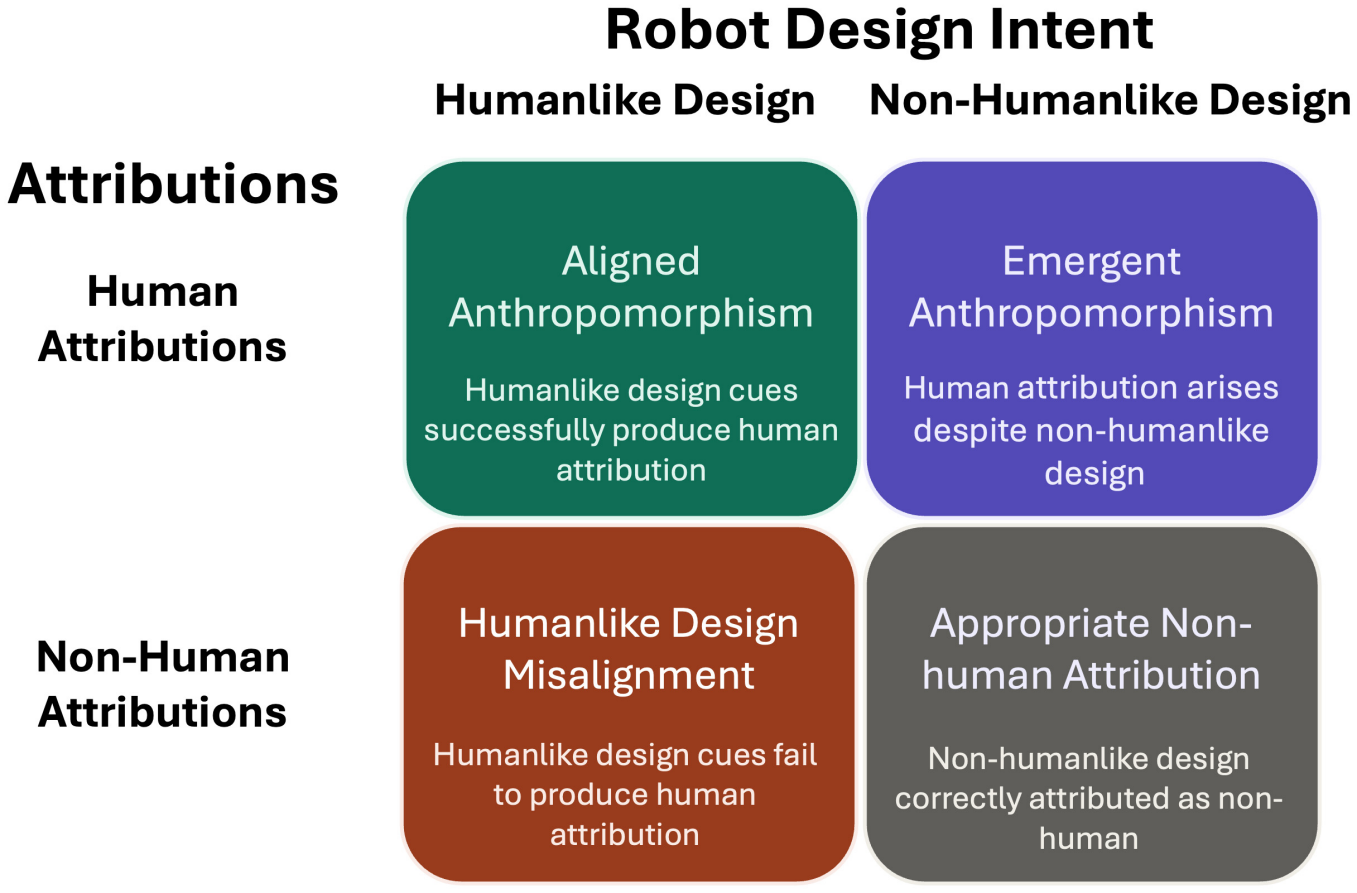
A framework for interpreting the relationship between humanlike robot design and human attribution. The horizontal dimension represents robot design intent and the vertical dimension represents how humans interpret that design. Crossing these dimensions yields four distinct outcomes. Aligned anthropomorphism occurs when humanlike design successfully produces human attribution. Emergent anthropomorphism occurs when human attribution arises despite non-humanlike design, driven by cognitive inference processes rather than design intent. Humanlike design misalignment occurs when humanlike design cues fail to produce human attribution. Appropriate non-human interpretation occurs when non-humanlike design is correctly interpreted as non-human.

To clarify the relationship between robot design and human interpretation of that design, we conceptualize the intersection of the two in Figure 3. The horizontal dimension represents whether the robot was designed to be humanlike and the vertical dimension represents whether humans attribute human characteristics and traits to that design. Crossing these dimensions yields four distinct interpretive outcomes that capture how the attribution of human traits and characteristics may align with or diverge from design properties. This framework makes explicit an important inferential boundary: human attributions are evidence about how people interpret a system, not necessarily direct evidence of the system's design-level humanlikeness. Each of the four outcomes below illustrates a different relationship between design intent and human interpretation.

#### 3.1.1 Aligned anthropomorphism

Aligned anthropomorphism occurs when robots designed with humanlike features lead users to attribute human characteristics, intentions, or mental states to the system. In this case, robot design intent and human anthropomorphism are aligned. Humanlike design cues successfully produce a human interpretation. For example, humanlike robots that incorporate facial features, gaze behavior, and social gestures often elicit attributions of mind, intention, or social awareness. Studies have shown that increasing the humanlike appearance of robots systematically increases the extent to which people attribute cognitive capacities, social traits, and emotions to them (Hegel et al., 2008; Zhao et al., 2019). In such cases, a human interpretation reflects the intended outcome of the robot's humanlike design.

#### 3.1.2 Emergent anthropomorphism

Emergent anthropomorphism occurs when robots not explicitly designed to be humanlike, nevertheless are attributed to have human traits and characteristics. In this case, human interpretation likely emerges more so from cognitive inference processes rather than from humanlike design. Even relatively simple machines or software systems can elicit human attributions when people interpret their behavior in intentional or social terms. For example, people frequently attribute intentions, emotions, or personalities to computers and automated systems despite their mechanical design (Nass and Moon, 2000). Similarly, users may interpret the behavior of mobile robots or algorithmic systems as goal-directed or intentional even when the system lacks humanlike form.

#### 3.1.3 Humanlike design misalignment

Humanlike design misalignment occurs when robots are intentionally designed with humanlike elements but people do not attribute human qualities to them. In these cases, the intended humanlike design cues fail to produce the expected attribution response. Misalignment may arise when humanlike cues are incomplete, inconsistent, perceptually ambiguous, or potential combined in ways that people do not like (Kim et al., 2022). In these situations, humanlike design may not successfully translate into humanlike perception.

#### 3.1.4 Appropriate non-human interpretation

Appropriate non-human interpretation occurs when robots designed as non-humanlike are interpreted accordingly. In these cases, perception accurately reflects the robot's non-humanlike design intent, and people do not attribute human characteristics to the system. For instance, zoomorphic robots such as Sony's Aibo are often interpreted as possessing animal characteristics or models of interaction that are similar to pets, or animal companions (e.g., needing training, for example, de Visser et al., 2022; Lum and Phillips, 2024). In these cases, non-human interpretations of the robot reflect successful alignment between robot design and human perception.

Together, these four outcomes illustrate that the relationship between humanlike design and human attribution is neither guaranteed nor uniform. Recognizing this variation is only possible, however, if humanlikeness as a design property and anthropomorphism as a psychological process are treated as distinct, and if humanlikeness itself is specified with sufficient precision to be meaningfully manipulated and measured. In practice, “humanlikeness” is often invoked as if it were self-evident or reducible to a single appearance continuum, yet the preceding discussion depends on the opposite assumption: that humanlike design is multi-dimensional, can be decomposed into identifiable cues, and can be manipulated in principled ways to selectively invite (or suppress) particular attributions.

## 4 Defining humanlikeness as a multidimensional design construct

To support conceptual clarity, we turn now to specifying what humanlikeness is as a design construct for robots and other autonomous machines, and to articulating the key considerations that delimit its scope, structure, and applicability for research and design. Although the term humanlike is widely used across human-robot interaction, human–computer interaction, and adjacent fields, it has often been invoked without explicit definition or with a hyper focus on appearance alone. In this section, we outline the explicit assumptions that underlie our construct definition as it applies to non-human machines.

*Humanlikeness is the degree to which an agent includes designed elements intended to mimic human characteristics, traits, features, processes, systems, and/or behaviors*. And there are several key points that motivate and delimit our definition offered here. First, this definition is grounded in a body of prior literature that distinguishes between robot humanlikeness as a property of design and anthropomorphism as a psychological process. And although the human–robot interaction research community has not always been consistent or explicit in maintaining this distinction, a number of scholars have nevertheless articulated and relied upon it in theoretically consequential ways. For example, Fink (2012) and others (Bhatti and Robert, 2023; DiSalvo et al., 2004, 2005; Eyssel et al., 2012; Fong et al., 2003; Rothstein et al., 2020) have described that an impactful way to influence the acceptance of social robots is through anthropomorphic (i.e., humanlike) design. And go on to clarify that humanlike design implies the humanlike parts of a robot's physical shape, the usage of facial expressions and other social cues, as well as natural humanlike interaction and communication patterns (e.g. speech, gaze, and gestures). Ho and MacDorman (2010) similarly described that “Objective humanness” the “human photorealism of a character's morphology, skin, texture, motion quality, or other formal property (p. 1511),” is different from attributions made to the agent. Foundational work on anthropomorphism by Guthrie (1997), likewise distinguishes between anthropomorphism–seeing objects or events as having some human characteristics–and actually having humanlike form.

Importantly, we intentionally define robot humanlikeness broadly, extending beyond outward physical appearance, which has received the greatest empirical attention to date. Although appearance is an important and salient kind of humanlikeness, restricting the humanlikeness construct to visual form alone risks overlooking other ways in which robot design can meaningfully mimic humans. Humanlikeness may vary along multiple dimensions, including the number, salience, and specificity of features; designed behaviors and interaction conventions; interface characteristics; and even the inspiration underlying processes of autonomy. For example, reinforcement learning as a machine learning process is inspired by associative learning processes in humans (i.e., operant conditioning) that first dominated the behaviorist tradition in human psychology. Its use thus represents a humanlike way for robots to learn. Broadening the construct in this way allows humanlikeness to capture important design decisions that shape interaction but are not immediately visible in morphological forms.

Extending the definition of humanlikeness beyond outward appearance also creates opportunities to identify systematic “first principles” of humanlike design. Prior work has already demonstrated the feasibility of such an approach for physical appearance. Phillips et al. (2018), for example, developed a bottom-up framework for characterizing robots' humanlike physical features by documenting and analyzing the presence of humanlike elements across the full-body appearance of hundreds of real-world robots. This work was the first to systematically define what constitutes a robot's humanlike physical appearance. They showed that perceptions of overall humanlike appearance can be systematically predicted by combinations of facial features, surface features (e.g., hair, skin, and apparel), and body structure and manipulators (e.g., arms, legs, and torso). As a result, this work created a systematic and parsimonious way to delineate and describe the exact way(s) in which any given robot's physical appearance resembles a human, addressing long-standing challenges related to experimental control and comparability across studies.

However, the work by Phillips et al. (2018) was limited to outward static physical appearance of robots. Because the design construct of humanlikeness can incorporate many ways in which robots mimic humans, additional research is needed to determine whether similar bottom-up approaches can be extended to other domains. For example, similar principles borrowed from domains like animation, dance and choreography (LaViers and Maguire, 2023; LaViers et al., 2018), or kinematics (Thompson et al., 2011) offer promising avenues for systematically describing humanlike movement. Prosody (Baird et al., 2018) could be used for voice, syntax selection and other linguistic features for language (Emnett et al., 2024; Lu et al., 2021) and so on. And additional work would be needed to detail how multiple dimensions of humanlikeness interact, how their aggregation shapes perceptions of overall humanlikeness, subsequent anthropomorphism, and downstream interaction outcomes. For instance, it is plausible that adding humanlike movement to a robot with relatively few humanlike physical features may contribute more strongly to perceptions of overall humanlikeness than appearance alone.

Finally, humanlikeness is inherently relational and perception-dependent. To describe a robot feature as humanlike presumes not only design intent, but also that people will perceive, experience, and interpret that a given humanlike feature as such. That is because there is no single objective standard human against which humanlike robots can be compared. Humans vary significantly in their appearance and similarity to one another. Yet, regardless of how much their appearance deviates from some common notion, they readily receive the same label as human. As a result, researchers who have operationalized forms of robot humanlikeness have treated the construct as a spectrum, from “less” to “more” and have relied on consensus in human judgment as a means to “objectively” quantify robot humanlikeness (Baird et al., 2018; Phillips et al., 2018). Empirical approaches that rely on consensus among human judgments to operationalize humanlikeness thus represent a pragmatic and theoretically grounded solution, even though they underscore the unavoidable role of human perception in defining the construct.

## 5 Humanlikeness in context: related and adjacent constructs

Having defined humanlikeness as a multidimensional design construct, the next step is to situate it within the broader conceptual space in which it is commonly discussed and interpreted. It is often invoked alongside a constellation of related constructs, some of which have been more formally theorized and empirically studied, and others more loosely or intuitively described. Each of the constructs listed below are used to describe machines, and how they resemble, evoke, or are interpreted as related to humans or other living things.

Accordingly, the following section maps the conceptual neighborhood surrounding humanlikeness. We include constructs that have received theoretical and empirical development across psychology, design, human-robot interaction or human-machine interaction literature broadly (e.g., animal-like and machinelike), as well as terms that lack formal conceptual grounding but nevertheless play an active role in how researchers and practitioners talk about machine similarity to humans (e.g., android and humanoid). By organizing and defining these concepts side by side, this section is intended to function as a theoretical reference that clarifies how humanlikeness relates to, overlaps with, and diverges from other commonly used terms, thereby supporting greater conceptual precision and cross-disciplinary communication. In this section, we briefly define several of these constructs and clarify their relationship to humanlikeness as a design property.

Table 1 presents a conceptual framework that organizes these constructs according to their ontological status (conditions, design properties, psychological processes, and representational practices), providing a structured overview of how humanlikeness and anthropomorphism are situated within a broader landscape of related concepts. The table is not intended to be exhaustive, nor to imply strict boundaries between constructs, but rather to offer a shared point of reference that supports clearer theorizing, more precise use of terminology, and improved comparability across research and design efforts.

### 5.1 Design forms

In work closely related to humanlikeness, DiSalvo et al. (2005, 2004) proposed a taxonomy of anthropomorphic design forms that helps articulate the many ways designed artifacts can incorporate humanlike elements, thus supporting the broad definition of humanlikeness. Through analyses of designed objects, they identified four distinct forms, each capturing a different way in which humanlike characteristics may be imitated in design.

Structural anthropomorphic form most closely aligns with the construct of humanlikeness as defined in this paper. It refers to design elements that imitate the structure or functioning of the human body, including shapes, volumes, mechanisms, or arrangements that resemble human anatomy or bodily operation. Gestural anthropomorphic form captures the imitation of human communicative movements, particularly non-verbal behaviors. Examples include motions such as nodding, shaking the head, or other gestures that convey meaning through culturally recognizable human actions. Anthropomorphic form of character represents a more abstract instantiation of humanlike design, in which artifacts imitate human traits, social roles, or functions embedded within broader societal conventions and contexts. This form is often used to convey identity, personality, or social meaning, such as signaling gendered or sexualized traits through design. Aware anthropomorphic form imitates human capacities for thought, intentionality, or inquiry. Commonly used in fiction, but also relevant to interactive technologies, this form suggests that an agent possesses humanlike awareness, including self-knowledge, social understanding, and the ability to reason about abstract concepts. As such, aware anthropomorphic form sits near the boundary between design features and psychological attribution and representation processes such as animism, personification, and related constructs discussed in later sections.

### 5.2 Anthropomorphic

The term anthropomorphic is an adjective derived from the Greek *Anthropos* meaning “human” and *Morph* meaning “shape or form,” and in its strictest etymological sense describes when something “has a human form (Oxford University Press, 2025).” Used in this narrow sense, anthropomorphic is closely related to humanlike as a descriptor of designed form. However, the term anthropomorphic also shares its root with anthropomorphism, the psychological process of attributing human qualities to non-human entities, and the two are frequently conflated in the literature. Accordingly, we prefer the term “humanlike” over “anthropomorphic” when describing robot design properties.

### 5.3 Android, gynoid, and humanoid

The Greek etymology of the term Android specifically refers to the qualities of male-ness or being “manlike.” Gynoid is the female equivalent, pertaining to the human female form, but is less commonly used and not found in dictionaries like the Oxford English or Merriam-Webster. The term humanoid is defined as, “with human form, having human characteristics,” as “an alien being having similar physical form to a human,” or as “an evolutionary precursor of humans (Oxford University Press, 2025)” In all three senses, these definitions are related to qualities of being humanlike either in form or development.

Android has taken on a more general meaning referring to robotic agents regardless of whether they represent the physical or socially constructed qualities of being male or female in sex or gender. English dictionary sources refer to androids as “An automation resembling a human being (Oxford University Press, 2025),” or “a mobile robot usually with human form (Merriam-Webster, 2025),” or “resembling a human being, esp. a man (rare, Oxford University Press, 2025).” In robotic engineering practice, the term android is also often reserved for robots that are very humanlike or very closely resembling specific humans (i.e., a copy) (Ishiguro, 2006b), either male or female (e.g., Geminoid and Kodomoroid). Some researchers have also considered humanoid robots and android robots as distinctly different, where the former refers to robots with both humanlike physical design qualities (hardware) and humanlike internal processing mechanisms (software), and the latter places heavy emphasis just on physical humanlikeness (Coradeschi et al., 2006; Ishiguro, 2006a).

The various definitions of the words, humanlike, android, and humanoid, each share commonalities with humanlikeness as they all refer to specifications of form with an emphasis on outward physical appearances and/or internal process resemblance to humans. Further, android and humanoid may refer to a specific point or range on the spectrum of humanlikeness. However, additional empirical evidence would be needed to support this hypothesis.

### 5.4 Animal-likeness

Similar to humanlikeness, animal-likeness as it is applied to machines has been considered a quality of design and form. In a similar manner as Phillips et al. (2018), researchers Löffler et al. (2020) found that robot animal-likeness is correlated with different constellations of animal-like features that include facial properties (i.e., face, eyes, head, mouth pupils, and childlike characteristics like an oversized head), surface properties (e.g., proportions, joints, color, plastic, gaps, texture, lights, hair, metal, and wheels), and animal-specific properties (i.e., skin, snout, tail, ears, familiarity, inspiration, legs, flexibility, and claws). In a bottom-up, feature based analysis, researchers found that the six best predictors of perceived overall animal-likeness of robots was the robots' joints, natural proportions, snout, color scheme, and use of plastic and metals in its fabrication.

The term zoomorphic which is defined as, “That [which] represents or imitates animal forms (Oxford University Press, 2025),” is synonymous with “animal-like.” And, similar to the distinctions between the constructs of anthropomorphism and humanlikeness, zoomorphism is the process of attributing animal qualities to non-animals which can include humans (Nanay, 2021), and animal-likeness and zoomorphic forms are characteristics of morphologies and designs.

Animal-like design is frequently employed by robot designers as a deliberate strategy for modulating the inference process, and to help human interaction partners to draw upon familiar interaction patterns (Kapalo et al., 2016; Klumpe et al., 2025; Krueger et al., 2021; Lum and Phillips, 2024; Phillips et al., 2016). By incorporating features that evoke living creatures without replicating specifically human form, designers can elicit social engagement, communication, and interdependence from people while limiting the degree to which fully human-specific attributions might be made or to avoid expectations that robots cannot meet. In accordance with our framework, this design logic is only legible within a framework that distinguishes design properties from psychological attributions. Further, the Haslam (2006) account of humanness includes traits that overlap with animals in the *human nature* dimension of humanness. And animalistic dehumanization occurs when people are likened to animals (see below). Thus, our framework treats animal-likeness as an important design construct relevant for mapping attributions to robot designs.

### 5.5 Machineness, machinelikeness, and robotlikeness

Definitions of the constructs of machineness and machinelikeness in the research literature are surprisingly limited, and offered definitions make it difficult to make clear distinctions between the two. Of this limited work, applicable is writing by Levy (2014) in discussions from the philosophy of sciences and biological sciences literature, and work by Haslam (2006) in describing mechanistic traits that are often ascribed to others in forms of mechanistic dehumanization. Dehumanization, its forms, and its relationships to humanness are discussed in much greater detail in later sections of this paper. But briefly, Haslam (2006) described that humanness, or the state of being human is composed of traits that are fundamentally human and characteristics that are uniquely human. When others are dehumanized, mechanistic traits are at times applied to others to do so. Mechanistic traits contrast human nature and include efficiency, regularity, automation-like rigidity, and conformity. Individuals who are considered mechanistic approach life (or are described as such) as unemotional, apathetic, and without spontaneity and they are considered fungible, passive, and their behavior lacking personal will. Haslam (2006) goes on to describe that these mechanistic traits are specifically used to deny or replace traits that are considered to be fundamentally human (i.e., human nature traits). As a result, those who are judged to have mechanistic traits are considered to be alien or foreign.

Dictionary definitions of machinelikeness mirror this description as characterized by regularity of action or stereotyped uniformity of product, regular movement or uniform patterns (Oxford University Press, 2025). In practical manipulations of robot behavior in laboratory studies, uniformity of robot behavior (i.e., neck and eye movements) have also been used as a manipulation of machinelikeness and in contrast to other manipulations of humanlikeness (Marchesi et al., 2021). Madhavan and Wiegmann (2007) in their work comparing trust in humans versus automation, described that trust in automation is evaluated based on expectations of perfection, invariance, and performance that is error-free, highly reliable, and consistent.

Further, Levy (2014) discusses machinelikeness as a way of describing how human systems, often in the biological sciences, are described as machinelike to aid understanding of how those systems work. A relevant example includes common descriptions of cell organelles where machine analogies are often used, e.g., “the mitochondria is the powerhouse of the cell.” Levy describes that machinelike systems are characterized by order and internal division which also include distinctness and interdependence among parts. Common to these descriptions and definitions of machinelikeness, however, is a focus on internal processes, systems, and functional traits as being machinelike.

In the physical sciences, machines are characterized by their ability to receive an input amount of energetic work and transfer that energy (often multiplied) to an output amount of work. However, it is currently unclear which, and the degree to which, other properties are encapsulated in the construct of machinelikeness as it is applied to agents. For example, based on the current state of the literature, it remains unclear what it means for an object to appear machinelike and what fundamental features and characteristics would underlie human perceptions of the machinelike appearance of agents. Often manipulations of machinelikeness in studies have relied on researcher intuition and have included selecting a few robot exemplars as “mechanical” representations. But unlike humanlikeness and animal-likeness the existing literature provides little principled guidance on what it means for an object to be machinelike in its design.

Recent work by Correia et al. (2024) offers a preliminary step toward characterizing what robotlike traits look like from the perspective of human perceivers. Working from a post-humanist theoretical tradition, Correia et al. (2024) proposed the concept of *robomorphism*, defined as the attribution of robot traits to non-robot entities, and produced an initial inventory of *robomorphic traits* through empirical workshop methods. Their inventory organized robot traits into three categories: physical-related traits (e.g., geometric and boxy physical form), action-related traits (e.g., non-fluid mechanical movement, replicable precision, and autonomy), and interaction-related traits (e.g., goal-directedness and sociability). Further, they define robotomorphic as characteristics, features, or qualities found in robotic entities and robotomorphism as the process of attributing robot traits to non-robot entities, including humans, animals, or objects, based on perceived similarity to robots.

Critically, Correia et al. (2024) concluded that, “the extensive identification of robomorphic traits remains underexplored” and that, “we lack terminology centered on the non-human aspects of a robot.” Although robomorphism is concerned with attributions directed toward non-robotic entities rather than with perceptions of robots themselves, this work nonetheless represents an important and convergent starting point for understanding what characteristics people associate with robots, and we encourage future work to build on it in developing a more principled account of machinelike and robotlike appearance and design.

To aid in future studies of machinelikeness of agents we offer two definitions based on the limited prior work, but also drawing inspiration from the definitions of humanness and humanlikeness. Machineness is “The condition of being machine including traits that are considered typical or common to machines but may also be shared directly, via analogy, or in contrast to biological entities like humans, including orderliness, efficiency, rigidity, conformity, passiveness, fungibility, deliberative, regularity, uniformity, differentiation and decomposition of parts, and relational interdependence of parts.” Like the distinctions between humanness and humanlikeness, the construct of machineness encompasses the condition of being machine, and the characteristics and traits that underlie that state of being. Whereas, machinelikeness includes designed characteristics that convey what is machine. However, at this time, more theoretical and empirical work is needed to appropriately validate and distinguish between these constructs.

## 6 Anthropomorphism is a psychological attribution process

We turn now to anthropomorphism as the second core construct in our framework. Because we argue that humanlikeness can function as a design-based facilitator of anthropomorphism, not only in general, but in shaping which human qualities are attributed to robots, it is necessary to clearly define anthropomorphism and situate it within its established conceptual and theoretical context (see also Table 1).

Anthropomorphism is an inductive psychological process by which humans make attributions about the qualities and characteristics of non-humans (Epley et al., 2007; Fink, 2012; Tondu and Bardou, 2009). Epley et al. (2007) provided one of the most used definitions which describes the process as, “The attribution of human properties, characteristics, or mental states to real or imagined non-human agents or objects (p. 865).” At its core, anthropomorphism describes characteristic patterns of default human thinking about non-human agents. And it can be predicted by motivational and cognitive determinants which include elicitation of agent self-knowledge, effectance motivation, and sociality.

### 6.1 Elicitation of self-knowledge

Agent self-knowledge is the primary cognitive determinant of anthropomorphism and refers to the “readily accessible,” “default concept,” or base knowledge used to reason about another, lesser-known agent. For humans, knowledge of the self is rich and formed early in development. Anthropomorphism is more likely to occur if a target agent increases the activation of a person's self-knowledge (or the “human” concept more generally) and that knowledge is applied to toward the non-human agent.

Morphological similarity to humans is a powerful way to increase the accessibility of such knowledge (e.g., Eddy et al., 1993; Johnson et al., 1998; Morewedge et al., 2007). And, other forms of agent similarity to humans, like voice (Eyssel et al., 2012; Muralidharan et al., 2014), use of gestures (Salem et al., 2011), and perceived ingroup membership (Eyssel and Kuchenbrandt, 2012) have all been shown to be ways in which anthropomorphism, specifically of robots, can be induced via self-knowledge elicitation.

### 6.2 Effectance

Effectance is one of two motivational determinants of anthropomorphism. It refers to the desire to effectively interact with other agents and humans' need to feel competence in doing so. Making the actions of non-humans understandable in the present and predictable in the future underlies humans' effectance motivation. Epley et al. (2007) described that anxiety surrounding uncertainty with an agent and the perceived importance of predicting an agent's behavior are therefore predictive of the tendency to anthropomorphize. Effectance motivation's role in anthropomorphism can be both dispositional or situational. Those with traits that favor feeling in control of their environment are more likely to anthropomorphize in times of uncertainty, and when uncertainty is induced (e.g., presented with technological malfunctions, unfamiliar, or unpredictable gadgets or even robots in general; Eyssel et al., 2011), anthropomorphism behaviors can be influenced (Waytz et al., 2010c).

### 6.3 Sociality

Sociality is the second motivational determinant of anthropomorphism and is based on the desire to establish social connections with other humans. By attributing humanlike qualities to non-humans, we create humanlike connections that can help to satisfy this need. Functionally, anthropomorphism can provide individuals with a sense of social connection by creating a source of social connection. It stands to reason then, that anthropomorphism would be more likely for individuals who feel a lack of social connection. And in fact, empirical studies have supported this hypothesis. Epley et al. (2008b) showed that participants who felt increasingly disconnected to others ascribed more socially supportive human traits (i.e., thoughtful, considerate, and sympathetic) to pets than participants who reported less disconnection. Additionally, when feelings of disconnection and loneliness were induced, people were more likely to make attributions and report believing in commonly anthropomorphized agents like God or angels (Epley et al., 2008a). And finally, reminding people of their social connections can also reduce the tendency to anthropomorphize (Bartz et al., 2016).

## 7 Anthropomorphism in context: related and adjacent constructs

### 7.1 Dehumanization

Dehumanization is the process by which human qualities are denied, rejected, or replaced by the representation of humans as non-human objects or animals. It is the opposite process of anthropomorphism, and denies human traits to human targets, often members of outgroups (Haslam, 2006). As the inverse process of anthropomorphism, the determinants of anthropomorphism (i.e., elicitation of agent self-knowledge, effectance, and sociality) when reversed, can trigger dehumanized perceptions of others. To illustrate, members of extreme outgroups like those experiencing homelessness often trigger very low levels of agent self-knowledge because they are often considered most dissimilar from one's self.

In neuroimaging studies, Harris and Fiske (2006) showed that when participants were thinking of members of these groups, brain regions associated with social cognition were less likely to be activated. Instead, these individuals triggered patterns of activation associated with disgust, suggesting that the unhoused individuals were more likely to be dehumanized by other perceivers.

Also, power dissimilarity between individuals can reduce effectance motivation (Waytz et al., 2010b). The need to effectively interact with others is diminished when one holds all the power in a given relationship, and studies have shown that those in positions of power are more likely to objectify subordinates (Gruenfeld et al., 2008). Those with strong socially cohesive ingroups are also less motivated to seek out additional social connections (i.e., sociality) from others, are more likely to see outgroups as different from their ingroup (Tajfel and Turner, 1986), are less likely to make mind attributions to others, and more likely to endorse dehumanizing violence like waterboarding and electric shock toward outgroup members (Waytz and Epley, 2012).

### 7.2 Infrahumanization

Similar to dehumanization, infrahumanization, sometimes called “subhumanization,” is the “Process by which people consider their ingroup as fully human and outgroups as less human and more animal-like (Leyens et al., 2007 p. 139).” Both dehumanization and infrahumanization are patterns of thinking that diminish or deny the unique qualities of humans to humans.

However, infrahumanization is considered a slightly subtler form of dehumanization. Where dehumanization is an outright denial of human uniqueness and nature to individuals; Infrahumanization, on the other hand, “strips outgroups of culture, civility, and higher moral functioning (p. 143),” and often involves ingroup members attributing fewer secondary emotions to outgroup members, which Leyens et al. (2007) argues distinguishes humans from animals (people do not differentiate primary emotions like anger, fear, and sadness between humans and animals). Infrahumanization is also a common and persistent phenomenon, existing even when ingroups have significant antagonism within themselves, and in the absence of conflict with outgroups (Haslam and Loughnan, 2014).

### 7.3 Humanness

Both dehumanization and infrahumanization reduce the perceived presence of traits and characteristics that define one's humanness. Both exist on a spectrum of humanness denial (Haslam, 2006). Defining what is denied in both of these processes requires an account of what exactly makes up one's humanness to be denied. Early work in infrahumanization gave some of the first empirical accounts of the defining features of humanness by trying to capture which characteristics were perceived to be unique to humans. Leyens et al. (2000) surveyed students, asking them to list uniquely human characteristics, finding themes of intelligence, reasoning, secondary emotions (called sentiments), and language. Further, Leyens et al. (2007) showed that people attribute fewer of these characteristics to outgroup members than ingroup members, which is a subtle denial of the members of the outgroup's humanness.

Haslam (2006) extended Leyens et al. (2000) work on humanness to account for the fundamental traits of humans in addition to characteristics unique to humans, “Characteristics that are typically or essentially human–that represent the concept's ‘core'–may not be the same ones that distinguish us from other species.” Thus, Haslam's framework defines humanness as composed of characteristics and traits that are both unique to humans (i.e., human uniqueness) and those that are essential to humans (i.e., human nature).

Thus, humanness is the condition of being human and is defined as two dimensional with *Human Uniqueness* composed of characteristics that distinguish humans from animals (e.g., civility, and social refinement, moral sensibility, rationality, and maturity) and *Human Nature* which includes the traits that are typical, fundamental, or common to being human but may also be shared with other species. These include positive and negative emotionality, warmth, openness, agency, depth, and flexibility considered fundamental to being human (Haslam et al., 2013).

Further, our perception of the characteristics that define human uniqueness occurs later in human development, is influenced more so by socialization, and tends to differ more cross-culturally than traits that define human nature. Because humanness is composed of two senses (human uniqueness and human nature), the denial of humanness (i.e., dehumanization) can also occur along both dimensions (Bain et al., 2009). The animalistic form of dehumanization often involves the denial of human uniqueness through the use of animalistic metaphors and the mechanistic form tends to focus on standardization, instrumentation, coldness and alertness and thereby the denial of human nature (Haslam, 2006).

### 7.4 Humanity (virtue)

Humanity as virtue can be thought of as moral standards and principles of conduct, like respect and benevolence, toward all persons as an end in and of itself. Meaning that, the treatment of individuals in a humane manner prohibits the manipulation and exploitation of individuals for selfish or even altruistic ends (Hill, 1980). In many legal contexts, it includes notions of humane treatment of persons and animals and provides many of the underlying principles of modern notions of human rights and the moral standing of humans and other living beings. In general, it refers to standards of treatment toward certain living beings simply because they are said beings, but does not necessarily require the unique qualities of humanness as proposed by Haslam (2006), as animals are often afforded humane treatment in a similar manner as humans.

### 7.5 Animism

Animism refers to attributing life to the non-living (Guthrie, 1997). It is an ontology in which objects and other non-human beings are said to possess life, for instance, souls, spirits, life force, or personhood. In the anthropological sciences, animism as a concept has been a foundational topic of study since the early inception of the field (Bird-David, 1999). Animism is often part of larger belief systems, for instance common to many indigenous peoples. Animism beliefs can include that animals, plants, rocks, rivers, weather, and human artifacts, among others are potentially alive and animated, and often possess agency with unique histories and even dispositional characteristics (Van Eyghen, 2023).

### 7.6 Personification

Personification refers to instances in which non-human things or abstractions–for instance, the cardinal sins–are represented as persons, commonly in literature and art, but also in other contexts like legal contexts, i.e., considering corporations as autonomous, self-directed economic beings (Mark, 1987), and marketing theory and strategy, i.e., managing corporate “image” and “identity” to consumers (Davies et al., 2001). It serves as a metaphorical communicative device (e.g., “Inflation is eating up our earnings”) that helps to highlight the salient features of a non-human concept (i.e., inflation is an adversary and is harmful, Dorst, 2011). Unlike anthropomorphism however, which has motivational determinants and can help facilitate one's understanding of unfamiliar entities, personification is largely used in rhetoric to facilitate analogical reasoning about abstract concepts and ideas.

### 7.7 Technomorphism (mechanomorphism)

Technomorphism (also known as mechanomorphism) is the attribution of technological characteristics to humans (Lum et al., 2011). Unlike mechanical forms of dehumanization, the purpose of technomorphism does not seem to be for denying humanness to others. Rather, it is often described as a tool for applying technological understanding as a way to better understand something about human thinking or behavior. For instance Lum et al. (2011) described that scientists, “Have used technomorphism to explain [for instance] how the human brain works by breaking it down into computer terms.” Technomorphism is also likely to share commonalities with the definition of machinelikeness offered by Levy (2014).

### 7.8 Ethopoeia

Although not commonly used across the HRI and HCI literature, Nass and Moon (2000) proposed ethopoeia as a dominant explanation for social responses to computers and other technological agents in the Computers as Social Actors (CASA) paradigm (Nass et al., 1994). Ethopoeia comes from an ancient Greek word used to describe the creation of a character as a rhetorical technique. Nass and Moon (2000) defined ethopoeia as, “a direct response to an entity as human while knowing that the entity does not warrant human treatment or attribution (Nass and Moon, 2000 p. 94).” The researchers describe that both anthropomorphism and ethopoeia constitute mindful responses toward non-humans.

## 8 Measurement resources

Having defined and situated humanlikeness, anthropomorphism, and their adjacent constructs within an integrated conceptual framework, we turn briefly to the question of measurement. As noted in Section 1.1, a practical goal of this paper is to support future empirical work by aggregating existing instruments that have been used to operationalize these constructs. Table 2 provides a summary of these instruments, organized by the construct they are primarily intended to capture. The measures and measurement tools (e.g., Anthropomorphic RoBOT Database) span humanlikeness as a design property, trait and state-based measures of anthropomorphism, machinelikeness, and technomorphism. Importantly, the table distinguishes between trait-based measures, which capture individual differences in anthropomorphism tendencies, and state-based measures, which assess responses to a specific agent or interaction context. Trait measures are more appropriate for examining individual difference moderators of anthropomorphism, whereas state measures are better suited to capturing the attributional outcomes of specific humanlike design choices.

This collection is intended to serve as a methodological resource for researchers. Although it is not definitively exhaustive, it provides a structured starting point for researchers seeking to operationalize these constructs in studies involving robotic agents and other non-human systems. Citations to source papers are included in Table 2 and full lists of items for each measure, along with administration instructions, response scales, and scoring procedures (when available) are provided in the Supplementary materials.

## 9 Implications

In this review, we aimed to clarify the distinction between humanlikeness as a design property and anthropomorphism as a psychological attribution process, and to articulate why maintaining this distinction matters for interdisciplinary research. A primary implication of this distinction is improved conceptual and methodological precision. Clear construct definitions allow researchers to describe more precisely what is being designed, manipulated, and measured, thereby improving communication across studies and disciplines. A shared conceptual language also supports cumulative science by enabling more meaningful comparison across findings and facilitating meta-analytic integration across bodies of literature.

To support this goal, we introduced a comprehensive definition of the humanlikeness construct as it applies to machines. We also placed humanlikeness and anthropomorphism in a broader conceptual framework (Table 1), and identified and curated existing measurement instruments and operational approaches in Table 2 and in the Supplementary materials, all of which should function as a practical resource for future empirical work.

A large portion of the discussion in this paper has also centered around the relationship between the design and implementation of humanlike features in robots and their consequences for anthropomorphism. Although much of this paper emphasizes how humanlike design features facilitate anthropomorphism attributions, the inverse relationship is also theoretically plausible. Individual differences in anthropomorphism tendency may shape how humanlike certain design cues are perceived in the first place. Although anthropomorphism is well established as a function of motivational and cognitive determinants (e.g., elicitation of self-knowledge, effectance motivation, and sociality; Epley et al., 2007; Waytz et al., 2010a), relatively little empirical work has examined how these determinants may also influence perceptions of humanlikeness itself. Understanding directionality and feedback between design, perception, and attribution remains an important avenue for future research.

At the same time, attributions could happen as a consequence of, in harmony with, or *despite* purposeful design choices. For the various robotics research communities, distinction between the two constructs is a necessary starting place for understanding how, when, and to what degree the (un)intentional design of humanlikeness allows people to “cross the gap” from design to anthropomorphism–from morphology to attribution, or from engineering to understanding.

Clarifying the distinction between humanlikeness and anthropomorphism also establishes important inferential boundaries for empirical interpretation. Attributions can provide evidence about how people perceive, interpret, and reason about robotic systems. They therefore support claims about human cognition, attribution processes, and interaction dynamics. However, such responses do not by themselves establish that a robot possesses humanlike design characteristics or that particular design features caused those attributions. Stronger claims about robot humanlikeness or design mechanisms require independent specification and measurement of design features, moderators, and downstream interaction outcomes, and ultimately better specification of these constructs.

Clarifying these constructs also carries normative and societal significance. Definitions and naming practices are not neutral: perceiving a non-human entity as possessing human or uniquely human traits may invite expectations of moral standing, responsibility, or care (Gray et al., 2007, 2012; Kim et al., 2025). As robots increasingly adopt humanlike features across physical, behavioral, and social domains, longstanding questions concerning moral consideration, responsibility, and rights are likely to intensify. These concerns are already reflected in debates surrounding robot rights (de Graaf et al., 2021; Gunkel, 2018), moral responsibility and use (Coeckelbergh, 2021), intimate and sexual relationships with robots (Frank and Nyholm, 2017; Nyholm and Frank, 2019), and real-world cases in which robots have been symbolically granted legal or civic status (Stone, 2017).

The implications of humanlikeness as a design property may also extend beyond dyadic human–robot interaction contexts to ones in which robotic or virtual agents mediate interactions between other humans. To illustrate, Brogi et al. (2024) developed a metaverse system that blended humans communicating through digital avatars with physical robot arms (i.e., “avatarms”) which interacted with the physical world in which one of the humans was located. They found that including the arm increased the feelings of presence among the remote humans. As robots and artificial agents occupy an increasingly central role as mediators of human-human interaction–not merely as targets of it–how their design properties are perceived, and what attributions they invite, become consequential not only for human–robot interaction dynamics but for the social and moral texture of human interactions as well (Gratch and Fast, 2022).

Increasing the humanlikeness of artificial agents may challenge existing conceptual boundaries altogether. Humanness itself has been theorized as context-dependent and historically contingent, shaped by collective social evolution rather than fixed biological criteria (Porra et al., 2020). As humans continue to evolve alongside technologies that increasingly mimic, extend, or augment human capabilities, what counts as human, humanlike, or machinelike may shift accordingly. Although terms such as cyborg have been used to describe technologically augmented humans (Oxford University Press, 2025), such language remains underdeveloped for describing everyday human-machine partnerships that “extend the reach of humans” in both literal and figurative ways. As these hybrids become commonplace, new conceptual categories may be required. Clarifying foundational constructs such as humanlikeness and anthropomorphism is therefore a necessary step toward responsibly navigating the social, ethical, and design implications of increasingly humanlike technologies.

## 10 Conclusions and contributions

This paper distinguishes humanlikeness as a multi-dimensional design construct from anthropomorphism as a psychological attribution process, clarifying a long-standing conceptual conflation across bodies of interdisciplinary research. Treating these constructs as analytically distinct enables more precise reasoning about their interaction and supports systematic links between design choices and attribution outcomes. To support this goal, we clarify and situate both constructs within an integrated conceptual framework and direct readers to established measurement instruments. Together, this work provides a foundation for more principled, context-sensitive approaches to humanlike robot design and evaluation.
