# Supplementary material for: Humanlikeness as design, anthropomorphism as inference: a conceptual framework for human–robot interaction
Source: Front Cognit. 2026 Jun 3;5:1786256. doi: 10.3389/fcogn.2026.1786256 (PMC13271121; doi:10.3389/fcogn.2026.1786256)
Supplement: Supplementary file 1 [file Data_Sheet_1.pdf]

frontiers

Humanlikeness as Design, Anthropomorphism as Inference: A Conceptual Framework for Human–Robot Interaction

1 SUPPLEMENTARY MATERIALS

The following are supplementary materials for the article titled, "Humanlikeness as Design, Anthropomorphism as Inference: A Conceptual Framework for Human–Robot Interaction" by Elizabeth K. Phillips and Ewart J. de Visser.

Anthropomorphism of robotic and other autonomous agents is of considerable interest across multiple research communities (e.g., human–robot interaction, human–computer interaction, social robotics, autonomous systems, human factors). How people perceive and subsequently interact with robotic designs has important implications for how those systems are used, misused, disused, or ultimately adopted and incorporated into human environments Parasuraman and Riley (1997).

The present Supplementary Materials are intended to serve as a methodological resource for researchers studying these phenomena. In the sections that follow, we aggregate and present a set of psychometric instruments and measurement techniques that have been used to quantify humanlikeness of robots, anthropomorphism towards them, and other closely related constructs. Although this collection is not intended to be exhaustive, it provides a structured starting point for researchers seeking to operationalize these constructs in studies involving robotic agents and other nonhuman systems. Importantly, we include both trait-based measures, which capture individual differences in anthropomorphic tendencies, and state-based measures, which assess context- or agent-specific anthropomorphism responses.

A key motivation for assembling these measures into a single document is the persistent conceptual and operational conflation of anthropomorphism with humanlikeness in the empirical literature. Anthropomorphism is most commonly defined as the attribution of human mental states, emotions, intentions, or agency to nonhuman entities, whereas humanlikeness more broadly refers to properties of design intended to mimic humans. Despite this distinction, prior research has frequently treated these constructs interchangeably. This practice complicates theoretical interpretation, limits comparability across studies, and obscures the psychological mechanisms underlying observed effects.

To help address this issue, the measures included here span distinct but related dimensions, including trait attribution, perceived humanness, technomorphic tendencies, and broader social and evaluative judgments of robotic and other machine agents. Where possible, relevant citations, full item lists, response formats, and scale structures are provided to support transparent reporting, replication, and informed instrument selection.

By collocating these instruments and making their conceptual targets explicit, this Supplementary Materials document is intended to support more precise construct operationalization and to serve as a tool for future researchers interested in these topics.

Please note that these measures are organized to match the order in which they are presented in Table 2 in the main manuscript.

2 ANTHROPOMORPHIC ROBOT (ABOT) DATABASE

The Anthropomorphic roBOT (ABOT) Database provides an empirically grounded framework for quantifying *robot humanlikeness as an appearance-based construct*, distinct from anthropomorphism as a psychological process of trait attribution. ABOT reports feature-presence scores for 16 humanlike appearance features (e.g., eyes, face, arms, legs), defined as the proportion of participants who judged each feature to be present for a given robot. These scores capture the perceptual salience of individual humanlike features without presuming human mental, emotional, or moral attributions.

Using these feature-presence scores, Phillips et al. (2018) identified four coherent appearance components via principal components analyses, reflecting the systematic co-occurrence of humanlike features (e.g., surface features, body manipulators, facial features). ABOT reports both principal component scores and corresponding dimension scores, with the latter providing a more interpretable summary of how strongly a robot embodies each appearance dimension. Dimension scores correlate highly with their associated principal component scores ($r > .92$). In addition, ABOT returns feature-level, component-level, and dimension-level *appearance profiles* for each robot, enabling researchers to visualize and compare how different robots instantiate distinct configurations of humanlike appearance features rather than relying on a single summary metric.

Finally, ABOT provides an empirically derived overall humanlikeness score for each robot, demonstrating that perceived humanlikeness is a *multidimensional construct* that can be predicted from constituent appearance features and their systematic groupings. Regression models show that appearance dimensions jointly account for a substantial proportion of variance in perceived humanlikeness ($R^2 > 80\%$), and these models can be used to estimate humanlikeness for robots not currently included in the database by specifying the presence or absence of humanlike features. All materials and tools are freely available at `http://www.abotdatabase.info`. Figure S1 depicts the profile of humanlikeness, feature, and dimension scores provided for the Nao robot in the ABOT database. For more specific details about the creation and validation of the ABOT Database see Phillips et al. (2018)

Additional researchers have used ABOT to extend classifications of robot zoomorphic design Löffler et al. (2020), robot genderedness and robot cuteness (Perugia et al. (2022, 2026)).

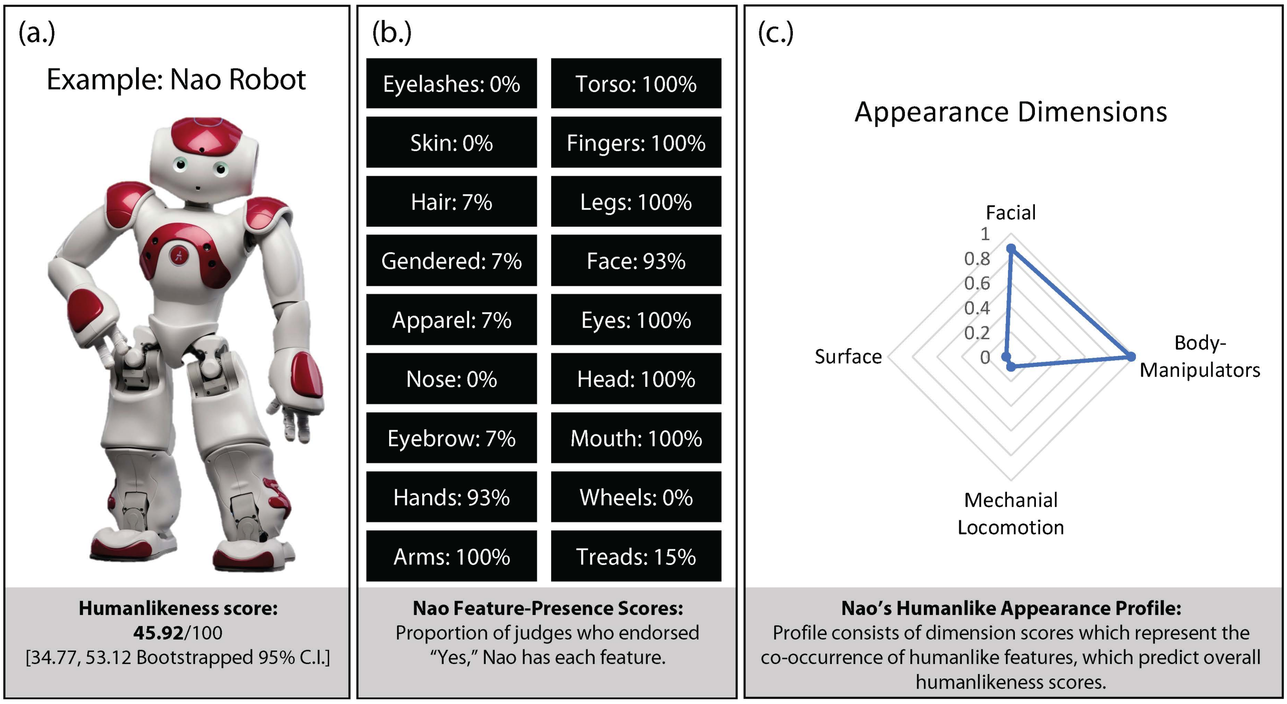

Figure S1: Profile of scores for the Nao robot provided by the ABOT Database. ABOT can be used to generate appearance profiles for robots, compare robots along specific humanlike feature dimensions, and/or estimate perceived humanlikeness for novel robot designs. Thus, ABOT can be used as a means to operationalize the humanlikeness of a robot while remaining conceptually distinct from measures of anthropomorphism. Panel (a) gives an example of the Nao robot with its empirically derived humanlikeness score. Panel (b) presents the "Feature presence" scores for the Nao robot, which represent the salience of each humanlike appearance feature. Feature presence scores were empirically derived from human judges and the percentages reported in Panel (b) represent the proportion of judges who endorsed that a given feature was indeed present on the Nao Robot. Panel (c) presents the humanlikeness "Dimension scores" which represent the systematic co-occurrence of features (derived from PCA analysis) into higher-order dimensions, where scores closer to 1 indicate that more of the defining features of that dimension are present on the Nao Robot than scores closer to 0. Panel (c) also represents the humanlike dimension "profile" for the Nao robot.

3 SINGLE-ITEM HUMANLIKENESS MEASURES

In some studies, a single item measure has been used to capture overall perceptions of robot physical humanlikeness when judging static images of robots (Phillips et al. (2018); Zhao et al. (2019)) as well as when examining the relationship acoustic features of synthesized speech and ratings of overall humanlikeness (Baird et al. (2018)).

In (Phillips et al. (2018)) and (Zhao et al. (2019)), participants were instructed to judge how much each entity in the photo looks like a human in its physical appearance. Then participants were shown an image of a robot (or entity) with the question, "Does this look physically humanlike?" Participants provided their judgment by dragging a slider to indicate where each robot falls on a continuum between "Not humanlike at all" (left-most point of the slider's scale) to "Just like a human" (right-most point of the slider's scale). The default position of the slider was set to the midpoint of the scale for each image. Participants' chosen slider position was then converted into a number ranging from 0 (Not humanlike at all) to 100 (Just like a human). Below is an image of the single-item measure and an example image participants have judged. In

Phillips et al. (2018) and Zhao et al. (2019), participants' humanlikeness scores were averaged by taking the mean of scores provided across participants.

In Baird et al. (2018) participants answered the question, "How close to human would you rate the voice speaking?" by using a 5-point Likert scale that ranged from 1= "Not at all", 5= "Extremely".

Figure S2 provides an example of the Alter robot and the single-item measure of humanlikeness used in Phillips et al. (2018).

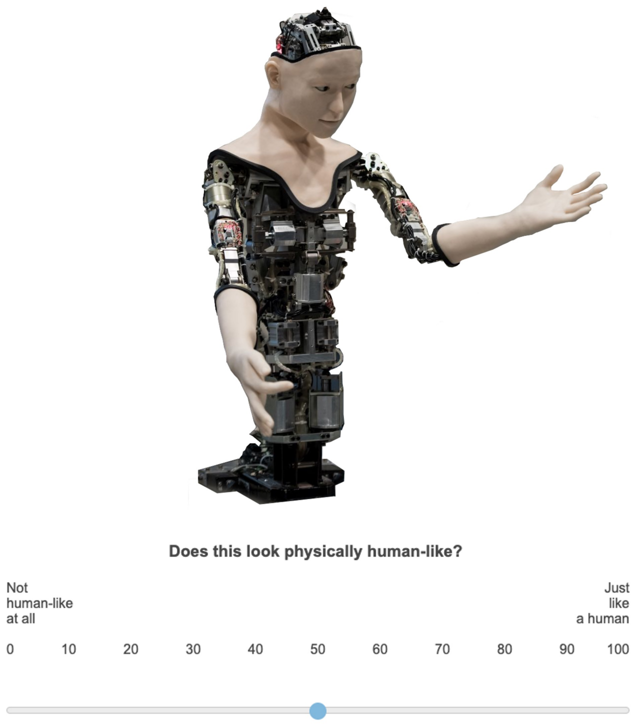

Figure S2: Alter Robot and example of single-item measure of robot humanlikeness. Image is available at www.abotdatabase.info

4 PERCEIVED HUMANNESS INDEX

The Perceived Humanness Index was developed by Ho and MacDorman (2010) and presented as an alternative to the Anthropomorphism subscale of The Godspeed Questionnaire (GSQ) and its specific use in studies of the Uncanny Valley Effect (i.e., the hypothesized relationship between the degree of an object's resemblance to a human and emotional responses to the object). Specifically, Ho and MacDorman (2010) found that the items composing the Anthropomorphism scale of the GSQ had poor discriminant validity for highly dissimilar robots (i.e., highly dissimilar robots had similar scores on the anthropomorphism items). They also stated that because many studies examining the effects of robot humanlike appearance are interested in psychological, social, and behavioral outcomes, it would be helpful to develop a subjective measure of an entity's humanlike appearance. The index includes 6 semantic differential scales where respondents rate an entity using two opposing poles. These include, artificial-natural, human-made–humanlike, without definite lifespan–mortal, inanimate–living, mechanical movement–biological movement, and synthetic–real. As part of measure development, the researchers also developed indices of perceived eeriness and attractiveness. These can be found in Ho and MacDorman (2010). The Perceived Humanness Index items are in Table S1. Ho and MacDorman (2010) did not provide explicit scoring instructions for this measure, however in Ho and MacDorman (2017) scores were averaged to obtain a composite perceived humanness score.

Table S1: Perceived Humanness Index items

**Left anchor**	**-3**	**-2**	**-1**	**0**	**1**	**2**	**3**	**Right anchor**
Artificial	-3	-2	-1	0	1	2	3	Natural
Human-made	-3	-2	-1	0	1	2	3	Humanlike
Without definite lifespan	-3	-2	-1	0	1	2	3	Mortal
Inanimate	-3	-2	-1	0	1	2	3	Living
Mechanical movement	-3	-2	-1	0	1	2	3	Biological movement
Synthetic	-3	-2	-1	0	1	2	3	Real

5 INDIVIDUAL DIFFERENCES IN ANTHROPOMORPHISM QUESTIONNAIRE (IDAQ)

The Individual Differences in Anthropomorphism Questionnaire (IDAQ) is a 30-item measure developed by Waytz et al. (2010b) and is a dispositional (i.e., trait-based) measure of anthropomorphism. The IDAQ measures stable individual differences in one's tendency to attribute humanlike traits to nonhumans. In a series of studies, Waytz et al. (2010a) illustrated that variability in an individual's tendency to anthropomorphize nonhuman targets is predictive of the degree to which those nonhuman targets are considered to possess secondary emotions, deserving of protection or harm, and worthy of trust, among others.

It includes items that cover three groups of entities (nonhuman animals, nature, and technology). The measure includes two subscales. The IDAQ subscale includes items that assess anthropomorphism (e.g., attributions of consciousness, freewill, intentions, etc.) and items in the IDAQ-NA subscale assess non-anthropomorphism attributions (e.g., physical and functional attributes like lethargy and usefulness). Respondents provide their response to each item using an 11-point scale that ranges from 0 (not at all) to 10 (very much). To score the IDAQ subscale: sum items 3, 4, 7, 9, 11, 12, 13, 14, 17, 20, 21, 22, 23, 26, and 29. To score the IDAQ-NA subscale: sum items 1, 2, 5, 6, 8, 10, 15, 16, 18, 19, 24, 25, 27, 28, and 30. Table S2 includes all of the IDAQ items. This measure has also been translated into Japanese, see Nakamura et al. (2024)

Table S2: Individual Differences in Anthropomorphism Questionnaire (IDAQ)

No.	Subscale	Item
1	IDAQ-NA	To what extent is the desert lethargic?
2	IDAQ-NA	To what extent is the average computer active?
3	IDAQ	To what extent does technology—devices and machines for manufacturing, entertainment, and productive processes (e.g., cars, computers, television sets)—have intentions?
4	IDAQ	To what extent does the average fish have free will?
5	IDAQ-NA	To what extent is the average cloud good-looking?
6	IDAQ-NA	To what extent are pets useful?
7	IDAQ	To what extent does the average mountain have free will?
8	IDAQ-NA	To what extent is the average amphibian lethargic?
9	IDAQ	To what extent does a television set experience emotions?
10	IDAQ-NA	To what extent is the average robot good-looking?
11	IDAQ	To what extent does the average robot have consciousness?
12	IDAQ	To what extent do cows have intentions?
13	IDAQ	To what extent does a car have free will?
14	IDAQ	To what extent does the ocean have consciousness?
15	IDAQ-NA	To what extent is the average camera lethargic?
16	IDAQ-NA	To what extent is a river useful?

Continued on next page

No.	Subscale	Item
17	IDAQ	To what extent does the average computer have a mind of its own?
18	IDAQ-NA	To what extent is a tree active?
19	IDAQ-NA	To what extent is the average kitchen appliance useful?
20	IDAQ	To what extent does a cheetah experience emotions?
21	IDAQ	To what extent does the environment experience emotions?
22	IDAQ	To what extent does the average insect have a mind of its own?
23	IDAQ	To what extent does a tree have a mind of its own?
24	IDAQ-NA	To what extent is technology—devices and machines for manufacturing, entertainment, and productive processes (e.g., cars, computers, television sets)—durable?
25	IDAQ-NA	To what extent is the average cat active?
26	IDAQ	To what extent does the wind have intentions?
27	IDAQ-NA	To what extent is the forest durable?
28	IDAQ-NA	To what extent is a tortoise durable?
29	IDAQ	To what extent does the average reptile have consciousness?
30	IDAQ-NA	To what extent is the average dog good-looking?

Note. Responses to each item are given on an 11-point scale ranging from 0 (*not at all*) to 10 (*very much*). IDAQ = anthropomorphism subscale (sum of items 3, 4, 7, 9, 11, 12, 13, 14, 17, 20, 21, 22, 23, 26, 29); IDAQ-NA = non-anthropomorphism attribution subscale (sum of items 1, 2, 5, 6, 8, 10, 15, 16, 18, 19, 24, 25, 27, 28, 30). Source: Waytz, Cacioppo, & Epley (2010).

6 INDIVIDUAL DIFFERENCES IN ANTHROPOMORPHISM QUESTIONNAIRE-CHILD FORM (IDAQ-CF)

Severson and Lemm (2016) extended the IDAQ by creating a child specific form (ages 5-9) for measuring individual differences in trait anthropomorphism in young children. The IDAQ-Child form (IDAQ-CF) is a 12-item measure of individual differences in anthropomorphism toward animals, nature, and technology. Severson and Lemm (2016) described that items related to free will in the original IDAQ were removed due to concerns with young children's comprehension of the concept of free will.

Child respondents indicate their agreement with the first question in the item by providing a Yes or "thumbs up," or No or "thumbs down" response. Then, respondents are asked to follow up with a response to the second question in the item using a 4-point scale, 0 (No), 1 (Yes-a little bit), 2 (Yes-a medium amount), 3 (Yes-a lot). To score the measure, the authors suggest computing the mean of the technology and nature items (8 items), and the mean of animal items (4 items) to compute two separate subscale scores. Table S3 includes the IDAQ-CF items.

Table S3. Individual Differences in Anthropomorphism Questionnaire—Child Form (IDAQ-CF)

Target	Subscale	Items
Technology	Intentions	Does a car do things on purpose? If yes, how much?
Technology	Emotions	Does a TV have feelings, like happy and sad? If yes, how much?
Technology	Consciousness	Does a robot know what it is? If yes, how much?
Technology	Mind	Does a computer think for itself? If yes, how much?
Nature	Intentions	Does the wind do things on purpose? If yes, how much?
Nature	Emotions	Does a mountain have feelings, like happy and sad? If yes, how much?
Nature	Consciousness	Does the ocean know what it is? If yes, how much?
Nature	Mind	Does a tree think for itself? If yes, how much?
Animals	Intentions	Does a turtle do things on purpose? If yes, how much?
Animals	Emotions	Does a cheetah have feelings, like happy and sad? If yes, how much?
Animals	Consciousness	Does a lizard know what it is? If yes, how much?
Animals	Mind	Does an insect or bug think for itself? If yes, how much?

Note. Child respondents first indicate agreement with each item using a Yes ("thumbs up") or No ("thumbs down") response. Respondents then answer the follow-up question using a 4-point scale: 0 (No), 1 (Yes—a little bit), 2 (Yes—a medium amount), 3 (Yes—a lot).

7 ANTHROPOMORPHIC TENDENCIES SCALE (ATS)

The Anthropomorphic Tendencies Scale (Chin et al. (2004, 2005)) is a 78-item measure also of individual differences in dispositions toward anthropomorphism. Items on the Anthropomorphic Tendencies Scale are similar to the IDAQ and the IDAQ-CF in that they contain items concerning anthropomorphism tendencies toward both animals and nature like pets and the ocean, and artifacts like computers, but also higher powers like luck or God. However, the items on the ATS focus on tendencies to engage in anthropomorphism behaviors towards nonhuman targets (e.g., "I would not yell at my computer if it did not function properly," "I treat a microwave like a human") rather than attributions of human qualities to nonhuman targets (e.g., "Does the average robot have consciousness?"). All items are rated on a 5-point Likert response scale ranging from 1 (Strongly disagree) to 5 (Strongly agree). Table S4 includes the ATS items. The authors do not provide explicit guidance on how to score this measure.

It is important to note that the IDAQ, IDAQ-CF, and the Anthropomorphic Tendencies Scale are measures designed to capture dispositional tendencies toward making human attributions toward nonhuman agents, which is conceptualized as an enduring trait that varies from individual to individual. These scales do not measure the cognitive and motivational determinants of anthropomorphism (e.g., elicitation of agent self-knowledge, effectance motivation, and sociality motivation), or the degree to which any one of these determinants are more impactful for engaging in anthropomorphism for a given individual over another. Rather, they measure one's predisposition towards anthropomorphism. To measure the degree to which the underlying psychological processes of anthropomorphism are engaged, one would need state-based measures of, for example, the degree which a given nonhuman agent has elicited human agent self-knowledge.

Table S4: Anthropomorphic tendencies scale items

Number	Item
1	When I am clearly upset, a COMPUTER does not know.
2	I do not act as if a COMPUTER has a spirit or life-force like people do.
3	A STUFFED TOY cannot communicate with people.
4	A COMPUTER does not have a personality like a person has a personality.
5	A MICROWAVE does not do things just to annoy me.
6	I would not praise a MICROWAVE when it does something I like.
7	When I am clearly upset, a MICROWAVE does not know.
8	An OCEAN does not do things just to annoy me.
9	I would not apologize to a BACKPACK for neglecting it.
10	I would not buy a present for a BACKPACK.
11	When I am clearly upset, a CAR does not know.
12	When I talk to a BACKPACK, I do not believe it understands me.
13	A STOMACH does not have a personality like a person has a personality.
14	An OCEAN does not have a personality like a person has a personality.
15	I do not act as if a MICROWAVE has a spirit or life-force like people do.

Continued on next page

Number	Item
16	A STUFFED TOY does not have a personality like a person has a personality.
17	A MICROWAVE has a spirit or life-force like people do.
18	I would not apologize to a COMPUTER for neglecting it.
19	I do not act as if a CAR has a spirit or life-force like people do.
20	When I talk to a HOUSE PLANT, I do not believe it understands me.
21	A MICROWAVE likes certain people better than others.
22	I do not act as if a HOUSE PLANT has a spirit or life-force like people do.
23	When I talk to a CAR, I do not believe it understands me.
24	I would not buy a present for a HOUSE PLANT.
25	I do not act as if LUCK has a spirit or life-force like people do.
26	I would not praise a STOMACH when it does something I like.
27	A STUFFED TOY is intelligent like a human is intelligent.
28	I do not act as if a STOMACH has a spirit or life-force like people do.
29	A BACKPACK does not do things just to annoy me.
30	I treat a MICROWAVE like a human.
31	If I were to get rid of a MICROWAVE, it would feel abandoned.
32	A MICROWAVE is intelligent like a human is intelligent.
33	A COMPUTER does not do things just to annoy me.
34	I would not praise a HOUSE PLANT when it does something I like.
35	If a HOUSE PLANT were to be destroyed, I would not mourn it like I would mourn the loss of a human.
36	If I were to get rid of a COMPUTER, it would feel abandoned.
37	I would not praise an INSECT when it does something I like.
38	A COMPUTER has a spirit or life-force like people do.
39	I would talk to a BACKPACK.
40	If a MICROWAVE were to be destroyed, I would not mourn it like I would mourn the loss of a human.
41	I treat a HOUSE PLANT like a human.
42	LUCK is intelligent like a human is intelligent.
43	An OCEAN cannot communicate with people.
44	When I talk to LUCK, I do not believe it understands me.
45	I would name a STOMACH.
46	I treat a COMPUTER like a human.
47	If a BACKPACK were to be destroyed, I would not mourn it like I would mourn loss of a human.
48	A HOUSE PLANT does not have a personality like a person has a personality.
49	A CAR has a spirit or life-force like people do.

Continued on next page

Number	Item
50	If a COMPUTER were to be destroyed, I would not mourn it like I would mourn the loss of a human.
51	A CAR does not do things just to annoy me.
52	LUCK cannot communicate with people.
53	I treat a BACKPACK like a human.
54	If I were to get rid of a BACKPACK, it would feel abandoned.
55	If I were to get rid of an INSECT, it would feel abandoned.
56	I would not apologize to an INSECT for neglecting it.
57	If I were to get rid of a HOUSE PLANT, it would feel abandoned.
58	A BACKPACK does not have a personality like a person has a personality.
59	LUCK does not have a personality like a person has a personality.
60	When I am clearly upset, a GOD OR HIGHER POWER does not know.
61	A GOD OR HIGHER POWER cannot communicate with people.
62	When I talk to a GOD OR HIGHER POWER, I do not believe it understands me.
63	I do not act as if a GOD OR HIGHER POWER has a spirit or life-force like people do.
64	I would not apologize to a GOD OR HIGHER POWER for neglecting it.
65	I would talk to a GOD OR HIGHER POWER.
66	I would not praise a GOD OR HIGHER POWER when it does something I like.
67	I would apologize to a GOD OR HIGHER POWER for accidentally hurting it.
68	A GOD OR HIGHER POWER has a spirit or life-force like people do.
69	A GOD OR HIGHER POWER is intelligent like a human is intelligent.
70	A GOD OR HIGHER POWER does not have a personality like a person has a personality.
71	I treat a GOD OR HIGHER POWER like a human.
72	I treat a PET like a human.
73	A MICROWAVE does not do things just to annoy me.
74	I do not act as if LUCK has a spirit or life-force like people do.
75	I would not buy a present for a BACKPACK.
76	If I were to get rid of a HOUSE PLANT, it would feel abandoned.
77	When I talk to a CAR, I do not believe it understands me.
78	I treat a MICROWAVE like a human.

Note. Respondents indicate their response to each item on a 5-point scale, 1 (Strongly disagree) to 5 (Strongly agree).

8 ANTHROPOMORPHISM QUESTIONNAIRE

Developed by Neave et al. (2015) this measure contains 20 items, 10 addressing childhood thoughts relating to toys, and 10 items addressing adult beliefs and behaviors. A total score can be calculated for each subscale, and a total score for the entire measure is obtained by summing the subscale totals. The maximum total score for the measure is 120. Table S5 includes the Anthropomorphism questionnaire items.

Table S5. Anthropomorphism questionnaire items

Number	Subscale	Item
1	Adult	I sometimes wonder if my computer deliberately runs more slowly after I have shouted at it.
2	Child	When I was a child I always made sure my favourite toy was comfortable (e.g., sitting up or tucked into bed) when I left the room.
3	Child	As a child I sometimes said, “hello” and “good night” to some of my favourite toys.
4	Child	When I was a child I held birthday parties for my favorite toys.
5	Adult	On occasions I feel that my computer/printer is being deliberately awkward.
6	Adult	Sometimes I wonder if my personal possessions appreciate it when I have given them a good clean.
7	Adult	On occasion I feel that weather conditions are being deliberately bad in order to ruin a social event.
8	Child	As a child when I put away my toys I made sure that any odd ones laying around were placed with the others so they wouldn’t feel lonely.
9	Adult	I don’t think certain cars have a specific personality.
10	Child	If I threw out a toy as a child I worried that it might think that I had rejected it.
11	Adult	If I accidentally break one of my favourite possessions I make sure that I apologize to it for my clumsiness.
12	Child	As a child I felt that some of my toys had become ill.
13	Adult	I think that some trees are friendly while others have an air of menace.
14	Adult	I sometimes think that if my computer/printer is made to feel happy and/or wanted, then they will be less likely to malfunction.
15	Child	As a child I felt at times that some of my toys were in a bad mood.
16	Child	As a child the thought of how my favourite toys would cope without me if I died was something that I worried about.
17	Adult	I sometimes feel that the sea can be angry.
18	Child	I sometimes wonder if toys are stored out of sight in a dark attic or room, they might feel lonely or unloved.
19	Adult	Part of the reason I picked a new car/electrical item was because when I first saw it I felt that it had a friendly personality.
20	Child	When I was a child I made sure that when I put my toys away the ones who were friends were placed side by side.

Note. Respondents indicate their response to each item on a 7-point scale, 0 (*Not at all*) to 6 (*Very much so*).

9 THE GODSPEED QUESTIONNAIRE

The Godspeed Questionnaire Series (GQS; called the Godspeed series having been translated into multiple languages) consists of five scales to evaluate the perception of social robots (Bartneck et al. (2009)). These scales are: Anthropomorphism, Animacy, Likeability, Perceived Intelligence, and Perceived Safety (Weiss and Bartneck (2015)). Respondents rate a robot on 24 different items using semantic differential scales. Semantic differentials have respondents choose between two opposing poles. It should be noted that the GSQ includes a number of subscales to measure not only anthropomorphism towards agents but also how much one likes the agent, and perceptions of the agent's mind (e.g., perceived intelligence). Additionally, the Anthropomorphism subscale includes items that characterize both the attribution of human characteristics (e.g., unconscious to conscious) as well ones related to the humanlike design of the robot (i.e., machine-like to humanlike). Table S6 includes the GSQ items. To score the GSQ take the mean of items scores in each subscale. Readers should note that Saad et al. (2026) reports that the GSQ lacks many of the necessary components needed to determine this scale's psychometric validity and Kaplan et al. (2021) notes that individual differences in persons' tendency to choose a rating on the measure showed the strongest relationship with their Godspeed scores, not the stimuli that were being evaluated. It is advisable that researchers using this scale check the reliability and validity of its scores.

Table S6: The Godspeed Questionnaire

Scale	Left anchor	1	2	3	4	5	Right anchor
**Anthropomorphism**	Fake	1	2	3	4	5	Natural
	Machinelike	1	2	3	4	5	Humanlike
	Unconscious	1	2	3	4	5	Conscious
	Artificial	1	2	3	4	5	Lifelike
	Moving rigidly	1	2	3	4	5	Moving elegantly
**Animacy**	Dead	1	2	3	4	5	Alive
	Stagnant	1	2	3	4	5	Lively
	Mechanical	1	2	3	4	5	Organic
	Artificial	1	2	3	4	5	Lifelike
	Inert	1	2	3	4	5	Interactive
	Apathetic	1	2	3	4	5	Responsive
**Likeability**	Dislike	1	2	3	4	5	Like
	Unfriendly	1	2	3	4	5	Friendly
	Unkind	1	2	3	4	5	Kind
	Unpleasant	1	2	3	4	5	Pleasant
	Awful	1	2	3	4	5	Nice
**Perceived Intelligence**	Incompetent	1	2	3	4	5	Competent
	Ignorant	1	2	3	4	5	Knowledgeable

Continued on next page

Scale	Left anchor	1	2	3	4	5	Right anchor
	Irresponsible	1	2	3	4	5	Responsible
	Unintelligent	1	2	3	4	5	Intelligent
	Foolish	1	2	3	4	5	Sensible
**Perceived Safety**	Anxious	1	2	3	4	5	Relaxed
	Agitated	1	2	3	4	5	Calm
	Quiescent	1	2	3	4	5	Surprised

Note. Participants rate their impression of the robot on each scale. To score the Godspeed Questionnaire, take the mean of item scores within each subscale. Source: Bartneck, Kulić, Croft, & Zoghbi (2009).

10 THE HUMAN-ROBOT INTERACTION EVALUATION SCALE

The Human–Robot Interaction Evaluation Scale (HRIES) is a 16-item measure intended to capture how people evaluate robots across trait attributions. The authors report that they adopted a multicomponent approach to anthropomorphism, explicitly integrating traits from social perception and dehumanization theory to better reflect the composite judgments people make when encountering robots. Respondents indicate their response to each item on a 7-point scale, 1 (Not at all) to 7 (Totally). For scoring the HRIES measure, the authors do not explicitly provide scoring guidance (e.g., mean, sum, median), but do report mean scores in their paper, and they do state that each dimension should be treated separately and scores should not be aggregated across the measure. Table S7 includes the HRIES items.

Table S7: Human-Robot Interaction Evaluation Scale

Number	Item	Subscale
1	Warm	Sociability
2	Likeable	Sociability
3	Trustworthy	Sociability
4	Friendly	Sociability
5	Alive	Animacy
6	Natural	Animacy
7	Real	Animacy
8	Humanlike	Animacy
9	Self-reliant	Agency
10	Rational	Agency
11	Intentional	Agency
12	Intelligent	Agency
13	Creepy	Disturbance
14	Scary	Disturbance
15	Uncanny	Disturbance
16	Weird	Disturbance

Note. Respondents indicate their response to each item on a 7-point scale, 1 (Not at all) to 7 (Totally). Note: It is recommended to randomize the presentation of items. If not possible, then randomize the presentation of factors.

11 ANTHROPOMORPHIC RESPONSE (AR) MEASURE

The Anthropomorphic Response (AR) measure was developed by Kim and Im (2023) and is a *state-based* measure of anthropomorphism designed to capture perceptions of and responses to AI agents during interaction. Kim and Im (2023) distinguish anthropomorphism response from prior definitions of anthropomorphism by emphasizing that it encompasses both perceptual and relational responses that arise specifically from human–nonhuman agent interaction, rather than from morphological features or stable individual tendencies alone.

The AR measure is a 10-item scale organized into five subscales: Connection, Helpfulness, Trust, Empathy, and Satisfaction. Respondents indicate their agreement with each item on a 7-point Likert scale ranging from 1 (*Strongly disagree*) to 7 (*Strongly agree*). It should be noted that several AR subscales, e.g., Helpfulness, Trust, and Satisfaction, overlap conceptually with outcomes commonly associated with anthropomorphism (e.g., trust, acceptance) rather than with the attribution process itself. Researchers are therefore encouraged to consider whether the full scale or specific subscales are most appropriate for their research questions. Table S8 includes the AR measure items.

The authors did not provide guidance on how to score this measure. Although not stated explicitly, their paper appears to present the mean of all of the items.

Table S8. Anthropomorphic Response (AR) measure items

**Construct**	**Item**	**Symbol**
Connection	I think the chatbot is responsive.	AR1
	I feel connected to the chatbot.	AR2
Helpfulness	I think the chatbot recognizes my needs.	AR3
	I think the chatbot is helpful.	AR4
Trust	I can trust the chatbot.	AR5
	I think the chatbot is reliable.	AR6
Empathy	I feel that the chatbot understands me.	AR7
	I feel comfortable sharing with the chatbot.	AR8
Satisfaction	I think I will use the chatbot again.	AR9
	I feel satisfied using the chatbot.	AR10

Note. Respondents indicate their agreement with each item on a 7-point Likert scale ranging from 1 (*Strongly disagree*) to 7 (*Strongly agree*).

12 MACHINE TRAIT SCALE

The Machine Trait Scale Kiesler and Goetz (2002) is a 105 item measure of perceptions of characteristic traits of machines. Participants rate 105 different adjectives on how machine-like they perceive each adjective using a 5-point scale ranging from 1 (Not machine-like) to 5 (Very machine-like). It may be applicable to measuring and validating the constructs of machineness and/or machinelikeness. However, more supporting empirical work is needed. Table S9 contains the Machine Trait Scale items. The authors did not provide instructions for scoring the measure.

Table S9: Machine Trait Scale Items

Number	Item	1	2	3	4	5
1	Talkative	1	2	3	4	5
2	Accurate	1	2	3	4	5
3	Curious	1	2	3	4	5
4	Energetic	1	2	3	4	5
5	Quarrelsome	1	2	3	4	5
6	Reliable	1	2	3	4	5
7	Tense	1	2	3	4	5
8	Breakable	1	2	3	4	5
9	Ingenious	1	2	3	4	5
10	Enthusiastic	1	2	3	4	5
11	Complicated	1	2	3	4	5
12	Confusing	1	2	3	4	5
13	Forgiving	1	2	3	4	5
14	Disorganized	1	2	3	4	5
15	Cumbersome	1	2	3	4	5
16	Worrisome	1	2	3	4	5
17	Unintelligent	1	2	3	4	5
18	Finds fault in others	1	2	3	4	5
19	Dangerous	1	2	3	4	5
20	Imaginative	1	2	3	4	5
21	Quiet	1	2	3	4	5
22	Dependable	1	2	3	4	5
23	Trustworthy	1	2	3	4	5
24	Dynamic	1	2	3	4	5
25	Lazy	1	2	3	4	5
26	Easy to manipulate	1	2	3	4	5
27	Emotionally stable	1	2	3	4	5

Continued on next page

Number	Item	1	2	3	4	5
28	Understandable	1	2	3	4	5
29	Inventive	1	2	3	4	5
30	Assertive	1	2	3	4	5
31	Works efficient	1	2	3	4	5
32	Aloof	1	2	3	4	5
33	Entertaining	1	2	3	4	5
34	Perseverant	1	2	3	4	5
35	Moody	1	2	3	4	5
36	Flexible	1	2	3	4	5
37	Fragile	1	2	3	4	5
38	Thorough	1	2	3	4	5
39	Quick	1	2	3	4	5
40	Artistic	1	2	3	4	5
41	Fun	1	2	3	4	5
42	Shy	1	2	3	4	5
43	Considerate	1	2	3	4	5
44	Handy	1	2	3	4	5
45	Informative	1	2	3	4	5
46	Calm	1	2	3	4	5
47	Intuitive	1	2	3	4	5
48	Routinized	1	2	3	4	5
49	Outgoing	1	2	3	4	5
50	Rude	1	2	3	4	5
51	Effortless	1	2	3	4	5
52	Has a lot of features	1	2	3	4	5
53	Follows through	1	2	3	4	5
54	Nervous	1	2	3	4	5
55	Depressed	1	2	3	4	5
56	Intricate	1	2	3	4	5
57	Plays with ideas	1	2	3	4	5
58	Cooperative	1	2	3	4	5
59	Easily distracted	1	2	3	4	5
60	Out of date	1	2	3	4	5
61	Comfortable	1	2	3	4	5
62	Musical	1	2	3	4	5
63	Powerful	1	2	3	4	5
64	Knowledgeable	1	2	3	4	5

Continued on next page

Number	Item	1	2	3	4	5
65	Irresponsible	1	2	3	4	5
66	Precise	1	2	3	4	5
67	Productive	1	2	3	4	5
68	Sensible	1	2	3	4	5
69	Original	1	2	3	4	5
70	Puzzling	1	2	3	4	5
71	Sturdy	1	2	3	4	5
72	Imaginative	1	2	3	4	5
73	Requires maintenance	1	2	3	4	5
74	Requires effort	1	2	3	4	5
75	Simple	1	2	3	4	5
76	Can save time	1	2	3	4	5
77	Tricky	1	2	3	4	5
78	Unreliable	1	2	3	4	5
79	Unstable	1	2	3	4	5
80	Reserved	1	2	3	4	5
81	User-friendly	1	2	3	4	5
82	Versatile	1	2	3	4	5
83	Interactive	1	2	3	4	5
84	Low-maintenance	1	2	3	4	5
85	Smart	1	2	3	4	5
86	Controllable	1	2	3	4	5
87	Helpful	1	2	3	4	5
88	Repetitive	1	2	3	4	5
89	Requires training	1	2	3	4	5
90	Ecologically good	1	2	3	4	5
91	Complex	1	2	3	4	5
92	Durable	1	2	3	4	5
93	High quality	1	2	3	4	5
94	Flawed	1	2	3	4	5
95	Careless	1	2	3	4	5
96	Portable	1	2	3	4	5
97	Could be improved	1	2	3	4	5
98	Safe	1	2	3	4	5
99	Sanitary	1	2	3	4	5
100	Clumsy	1	2	3	4	5
101	Cost efficient	1	2	3	4	5

Continued on next page

Number	Item	1	2	3	4	5
102	Hazardous	1	2	3	4	5
103	Heavy-duty	1	2	3	4	5
104	Specialized	1	2	3	4	5
105	Relaxed	1	2	3	4	5

Note. Respondents indicate how machine-like they perceive each adjective or descriptor to be on a 5-point scale from 1 (*Not machine-like*) to 5 (*Very machine-like*).

13 TECHNOMORPHIC TENDENCIES SCALE (TTS)

The technomorphic tendencies scale (TTS) is a 30-item scale to measure individual differences in the tendency to technomorphize (Lum et al. (2011)). It consists of two subscales. The first subscale includes items related to the degree to which respondents' tendency to use technomorphic schema for understanding agents. The second includes items about one's perceptions of others who exhibit technological characteristics (e.g., "A person who is wearing a Bluetooth device is computer-like"). Respondents use a 5-point Likert scale to respond ranging from 1 (Strongly disagree) to 5 (Strongly agree) to respond to each item. To score the TTS, take the mean of responses to all items. Table S10 includes the TTS items. The full scale can also be found in Appendix A.3 of Lum (2011), `https://stars.library.ucf.edu/cgi/viewcontent.cgi?article=2865&context=etd`.

Table S10: Technomorphic Tendencies Scale

Number	Item
1	I think of a machine with moving parts working in conjunction with one another when looking at how people dance.
2	I think of food as fuel that my body converts to energy in order to work as an effective machine.
3	I think of a computer with moving parts working in conjunction with one another when looking at how the human body moves.
4	I think of food as fuel that my body converts to energy in order to work as an effective computer.
5	I think of a machine with moving parts working in conjunction with one another to understand a concept such as how love works.
6	I use an analogy about how a machine works to understand a concept such as tiredness.
7	I think of a computer with moving parts working in conjunction with one another to understand a concept such as hatred.
8	I think of a computer with moving parts working in conjunction with one another to understand a concept such as how love works.
9	I break down the concept into machine-like parts when I am trying to understand how memory works.
10	I break down the concept into machine-like parts when I am trying to understand how an ecosystem works.

Continued on next page

**Number**	**Item**
11	I break down the concept into machine-like parts when I am trying to understand how a business works.
12	I break down the concept into computer-like parts when I am trying to understand how the human body operates.
13	I break down the concept into computer-like parts when I am trying to understand how a business works.
14	I think that a person who is wearing a Bluetooth device is machine-like.
15	I think that a person who is using a cochlear implant (hearing aid) is machine-like.
16	I think that a person who is using multiple technological devices at once is machine-like.
17	I think that a person who is wearing a Bluetooth device is computer-like.
18	I think that a person who has an artificial heart is computer-like.
19	I think that a person who has a prosthesis is computer-like.
20	I think that a person who is using multiple technological devices at once is computer-like.
21	I think that a person who has exceptional math skills is machine-like.
22	I think that a person who is detached during emotional discussions is machine-like.
23	I think that a person who uses precise vocabulary in casual situations is machine-like.
24	I think that a person who uses logic to answer questions is computer-like.
25	I think that a person who uses precise vocabulary in casual situations is computer-like.
26	I think that a person who uses his/her skills to improve negative situations is machine-like.
27	I think that a person who understands human emotions is machine-liked.
28	I think that a person who empathizes with others is machine-like.
29	I think that a person who solves complex problems is computer-like.
30	I think that a person who understands human emotions is computer-like.

Note. Responses to each statement are given on a 5-point scale, 1 (Strongly disagree) to 5 (Strongly agree).

REFERENCES

Baird, A., Parada-Cabaleiro, E., Hantke, S., Burkhardt, F., Cummins, N., and Schuller, B. (2018). The perception and analysis of the likeability and human likeness of synthesized speech. In *Proceedings of the 19th annual conference of the International Speech Communication Association (ISCA)*. 2863–2687. doi:10.21437/Interspeech.2018-1093

Bartneck, C., Kulić, D., Croft, E., and Zoghbi, S. (2009). Measurement instruments for the anthropomorphism, animacy, likeability, perceived intelligence, and perceived safety of robots. *Social Robotics*

Chin, M. G., Sims, V. K., Clark, B., and Lopez, G. R. (2004). Measuring individual differences in anthropomorphism toward machines and animals. In *Proceedings of the Human Factors and Ergonomics Society Annual Meeting* (SAGE Publications Sage CA: Los Angeles, CA), vol. 48, 1252–1255. doi:10.1177/154193120404801110

Chin, M. G., Yordon, R. E., Clark, B. R., Ballion, T., Dolezal, M. J., Shumaker, R., et al. (2005). Developing and anthropomorphic tendencies scale. In *Proceedings of the Human Factors and Ergonomics Society*

Annual Meeting (SAGE Publications Sage CA: Los Angeles, CA), vol. 49, 1266–1268. doi:10.1177/154193120504901311

Ho, C.-C. and MacDorman, K. F. (2010). Revisiting the uncanny valley theory: Developing and validating an alternative to the godspeed indices. *Computers in Human Behavior* 26, 1508–1518

Ho, C.-C. and MacDorman, K. F. (2017). Measuring the uncanny valley effect: Refinements to indices for perceived humanness, attractiveness, and eeriness. *International Journal of Social Robotics* 9, 129–139

Kaplan, A. D., Sanders, T. L., and Hancock, P. A. (2021). Likert or not? how using likert rather than biposlar ratings reveal individual difference scores using the godspeed scales. *International Journal of Social Robotics* 13, 1553–1562

Kiesler, S. and Goetz, J. (2002). Machine trait scales for evaluating mechanistic mental models of robots and computer-based machines. *Unpublished manuscript, Carnegie Mellon University* `https://www.cs.cmu.edu/~kiesler/anthropomorphism-org/pdf/Machine_scale.pdf`

Kim, J. and Im, I. (2023). Anthropomorphic response: Understanding interactions between humans and artificial intelligence agents. *Computers in Human Behavior* 139, 107512

Löffler, D., Dörrenbächer, J., and Hassenzahl, M. (2020). The uncanny valley effect in zoomorphic robots: The u-shaped relation between animal likeness and likeability. In *Proceedings of the 2020 ACM/IEEE international conference on human-robot interaction*. 261–270

Lum, H. C. (2011). Are we becoming superhuman cyborgs? how technomorphism influences our perceptions of the world around us

Lum, H. C., Sims, V. K., Chin, M. G., and Halse, S. E. (2011). Are we becoming super-human cyborgs? examination of technomorphism and the creation of a technomorphic tendencies scale. In *Proceedings of the Human Factors and Ergonomics Society Annual Meeting* (SAGE Publications Sage CA: Los Angeles, CA), vol. 55, 1370–1374

Nakamura, H., Matsuo, A., and Majima, Y. (2024). Development of the japanese version of the individual differences in anthropomorphism questionnaire. *The Japanese Journal of Psychology* 95, 190–200. doi:10.4992/jjpsy.95.22217

Neave, N., Jackson, R., Saxton, T., and Hönekopp, J. (2015). The influence of anthropomorphic tendencies on human hoarding behaviours. *Personality and Individual Differences* 72, 214–219

Parasuraman, R. and Riley, V. (1997). Humans and automation: Use, misuse, disuse, abuse. *Human factors* 39, 230–253

Perugia, G., Ankersmit, S., Jansen, N., Guidi, S., et al. (2026). The ’aww’factor: Robot cuteness as a catalyst for emotional responses and caretaking tendencies. In *Proceedings of the 21st ACM/IEEE International Conference on Human-Robot Interaction*. 687–696

Perugia, G., Guidi, S., Bicchi, M., and Parlangeli, O. (2022). The shape of our bias: Perceived age and gender in the humanoid robots of the abot database. In *2022 17th ACM/IEEE International Conference on Human-Robot Interaction (HRI)* (IEEE), 110–119

Phillips, E., Zhao, X., Ullman, D., and Malle, B. F. (2018). What is human-like?: Decomposing robots’ human-like appearance using the anthropomorphic robot (abot) database. In *Proceedings of the 2018 ACM/IEEE International Conference on Human-Robot Interaction* (ACM), 105–113

Saad, L., Roesler, E., Phillips, E., and Trafton, J. G. (2026). Choosing the ”perfect” scale: a primer to evaluate existing scales in hri. *ACM Transactions on Human-Robot Interaction* 15, 1–30

Severson, R. L. and Lemm, K. M. (2016). Kids see human too: Adapting an individual differences measure of anthropomorphism for a child sample. *Journal of Cognition and Development* 17, 122–141

Waytz, A., Cacioppo, J., and Epley, N. (2010a). Individual differences in anthropomorphism questionnaire. *Perspectives on Psychological Science*

Waytz, A., Cacioppo, J., and Epley, N. (2010b). Who sees human? the stability and importance of individual differences in anthropomorphism. *Perspectives on psychological science* 5, 219–232

Weiss, A. and Bartneck, C. (2015). Meta analysis of the usage of the godspeed questionnaire series. In *2015 24th IEEE International Symposium on Robot and Human Interactive Communication (RO-MAN)* (IEEE), 381–388

Zhao, X., Phillips, E., and Malle, B. F. (2019). How people infer a humanlike mind from a robot body. *PsyArXiv, preprint* Retrieved from osf.io/wksda
